# Supplementary material for: Solar-driven sugar production directly from CO2 via a customizable electrocatalytic–biocatalytic flow system
Source: Nat Commun. 2024 Mar 25;15:2636. doi: 10.1038/s41467-024-46954-w (PMC10963751; doi:10.1038/s41467-024-46954-w)
Supplement: Supplementary file 3 — Solar Cells Reporting Summary [file 41467_2024_46954_MOESM3_ESM.pdf]

nature portfolio

Corresponding author(s): Yujie Xiong

Solar Cells Reporting Summary

Nature Portfolio wishes to improve the reproducibility of the work that we publish. This form is intended for publication with all accepted papers reporting the characterization of photovoltaic devices and provides structure for consistency and transparency in reporting. Some list items might not apply to an individual manuscript, but all fields must be completed for clarity.

For further information on Nature Research policies, including our data availability policy, see Authors & Referees.

Experimental design

Please check the following details are reported in the manuscript, and provide a brief description or explanation where applicable.

1. Dimensions

Area of the tested solar cells

- [x] Yes — The illuminated area of the tested solar cell is 38.9 cm2.
- [ ] No — *Explain why this information is not reported/not relevant.*

Method used to determine the device area

- [ ] Yes — *Provide a description of the method and state where this information can be found in the text.*
- [x] No — The illuminated area of the tested solar cell is provided by a third-party certified test report (Supplementary Fig. 10).

2. Current-voltage characterization

Current density-voltage (J-V) plots in both forward and backward direction

- [x] Yes — The data of J-V plots is provided by a third-party certified test report (Supplementary Fig. 10).
- [ ] No

Voltage scan conditions

- [ ] Yes — *Provide a description of the measurement conditions (e.g. scan direction, speed, dwell times).*
- [x] No — The data of J-V plots is provided by a third-party certified test report.

Test environment

- [x] Yes — Temperature:24.9 ℃, relative humidity: 49%, atmosphere: air
- [ ] No — *Explain why this information is not reported/not relevant.*

Protocol for preconditioning of the device before its characterization

- [ ] Yes — *Provide a description of the protocol.*
- [x] No — The data of J-V plots is provided by a third-party certified test report.

Stability of the J-V characteristic

- [x] Yes — Before the experiment, the solar cell was exposed under the light source until the surface temperature stabilizing. The J-V plots were collected multiple times.
- [ ] No — *Explain why this information is not reported/not relevant.*

3. Hysteresis or any other unusual behaviour

Description of the unusual behaviour observed during the characterization

- [ ] Yes — *Provide a description of hysteresis or any other unusual behaviour observed during the characterization.*
- [x] No — There is no unusual behaviour observed during the characterization.

Related experimental data

- [ ] Yes — *Provide a description of the related experimental data.*
- [x] No — There is no unusual behaviour observed during the characterization.

4. Efficiency

External quantum efficiency (EQE) or incident photons to current efficiency (IPCE)

- [x] Yes — The used photovoltaic cell has a certificated solar-to-electricity efficiency of 15.5% (Supplementary Fig. 10, Supplementary Table 1).
- [ ] No — *Explain why this information is not reported/not relevant.*

A comparison between the integrated response under the standard reference spectrum and the response measure under the simulator

- [ ] Yes — *State where this information can be found in the text.*
- [x] No — All tests are carried out under AM 1.5G simulated sun light.

For tandem solar cells, the bias illumination and bias voltage used for each subcell

☐ Yes
☒ No

Provide a description of the measurement conditions.

We used a uniform light modulator to ensure the bias illumination of each subcells are equal.

5. Calibration

Light source and reference cell or sensor used for the characterization

☒ Yes
☐ No

The Light is provided by a solar simulator and the reference cell is a WPVS monocrystalline silicon reference solar cell

Explain why this information is not reported/not relevant.

Confirmation that the reference cell was calibrated and certified

☒ Yes
☐ No

The reference cell was certified by National Institute of Metrology.

Explain why this information is not reported/not relevant.

Calculation of spectral mismatch between the reference cell and the devices under test

☒ Yes
☐ No

The data is provided by a third-party certified test report (Supplementary Fig. 10).

Explain why this information is not reported/not relevant.

6. Mask/aperture

Size of the mask/aperture used during testing

☐ Yes
☒ No

Report the size of the mask/aperture.

No mask/apeture were used during tests.

Variation of the measured short-circuit current density with the mask/aperture area

☐ Yes
☒ No

Report the difference in the short-circuit current density values measured with the mask and aperture area.

No mask/apeture were used during tests.

7. Performance certification

Identity of the independent certification laboratory that confirmed the photovoltaic performance

☒ Yes
☐ No

Fujian Metrology Institute.

Explain why this information is not reported/not relevant.

A copy of any certificate(s)

☒ Yes
☐ No

Certificate copies are provided in Supplementary Fig. 10.

Explain why this information is not reported/not relevant.

8. Statistics

Number of solar cells tested

☒ Yes
☐ No

One.

Explain why this information is not reported/not relevant.

Statistical analysis of the device performance

☒ Yes
☐ No

The used photovoltaic cell has a certificated solar-to-electricity efficiency (Supplementary Fig. 10, Supplementary Table 1). The J-V plots were collected multiple times.

Explain why this information is not reported/not relevant.

9. Long-term stability analysis

Type of analysis, bias conditions and environmental conditions

☒ Yes
☐ No

We have perform the stability test for 120 hours for our PV-EC system and given the stability data (Fig. 2e, Supplementary Fig. 11). Room temperature, in air.

Explain why this information is not reported/not relevant.
