# Supplementary material for: Solar-driven sugar production directly from CO2 via a customizable electrocatalytic–biocatalytic flow system
Source: Nat Commun. 2024 Mar 25;15:2636. doi: 10.1038/s41467-024-46954-w (PMC10963751; doi:10.1038/s41467-024-46954-w)
Supplement: Supplementary file 4 — Reporting Summary [file 41467_2024_46954_MOESM4_ESM.pdf]

nature portfolio

Corresponding author(s): Yujie Xiong

Last updated by author(s): Feb 29, 2024

Reporting Summary

Nature Portfolio wishes to improve the reproducibility of the work that we publish. This form provides structure for consistency and transparency in reporting. For further information on Nature Portfolio policies, see our Editorial Policies and the Editorial Policy Checklist.

Statistics

For all statistical analyses, confirm that the following items are present in the figure legend, table legend, main text, or Methods section.

n/a	Confirmed	
☐	☒	The exact sample size (*n*) for each experimental group/condition, given as a discrete number and unit of measurement
☐	☒	A statement on whether measurements were taken from distinct samples or whether the same sample was measured repeatedly
☒	☐	The statistical test(s) used AND whether they are one- or two-sided<br>*Only common tests should be described solely by name; describe more complex techniques in the Methods section.*
☒	☐	A description of all covariates tested
☒	☐	A description of any assumptions or corrections, such as tests of normality and adjustment for multiple comparisons
☐	☒	A full description of the statistical parameters including central tendency (e.g. means) or other basic estimates (e.g. regression coefficient) AND variation (e.g. standard deviation) or associated estimates of uncertainty (e.g. confidence intervals)
☒	☐	For null hypothesis testing, the test statistic (e.g. *F*, *t*, *r*) with confidence intervals, effect sizes, degrees of freedom and *P* value noted<br>*Give P values as exact values whenever suitable.*
☒	☐	For Bayesian analysis, information on the choice of priors and Markov chain Monte Carlo settings
☒	☐	For hierarchical and complex designs, identification of the appropriate level for tests and full reporting of outcomes
☒	☐	Estimates of effect sizes (e.g. Cohen's *d*, Pearson's *r*), indicating how they were calculated

Our web collection on statistics for biologists contains articles on many of the points above.

Software and code

Policy information about availability of computer code

Data collection	No software was used.
Data analysis	Statistical analysis. All the statistical analyses were performed using Origin 2021, Design Export,Excel 2019 and Nano Measurer software. Statistical comparisons were carried out by performing two-sided Student's t-test.

For manuscripts utilizing custom algorithms or software that are central to the research but not yet described in published literature, software must be made available to editors and reviewers. We strongly encourage code deposition in a community repository (e.g. GitHub). See the Nature Portfolio guidelines for submitting code & software for further information.

Data

Policy information about availability of data

All manuscripts must include a data availability statement. This statement should provide the following information, where applicable:

- Accession codes, unique identifiers, or web links for publicly available datasets
- A description of any restrictions on data availability
- For clinical datasets or third party data, please ensure that the statement adheres to our policy

The authors declare that all data supporting the findings of this study are available in the article and its Supplementary Information. Source data are provided with this paper.

Research involving human participants, their data, or biological material

Policy information about studies with human participants or human data. See also policy information about sex, gender (identity/presentation), and sexual orientation and race, ethnicity and racism.

Reporting on sex and gender	Not Relevant.
Reporting on race, ethnicity, or other socially relevant groupings	Not Relevant.
Population characteristics	Not Relevant.
Recruitment	Not Relevant.
Ethics oversight	Not Relevant.

Note that full information on the approval of the study protocol must also be provided in the manuscript.

Field-specific reporting

Please select the one below that is the best fit for your research. If you are not sure, read the appropriate sections before making your selection.

☐ Life sciences ☐ Behavioural & social sciences ☒ Ecological, evolutionary & environmental sciences

For a reference copy of the document with all sections, see nature.com/documents/nr-reporting-summary-flat.pdf

Ecological, evolutionary & environmental sciences study design

All studies must disclose on these points even when the disclosure is negative.

Study description	We report a hybrid electrocatalytic–biocatalytic flow system, coupling photovoltaics-powered electrocatalysis (CO2 to formate) with five enzyme cascade platform (formate to sugar) engineered via genetic mutation. This flow system can in principle be programmed by coupling with diverse enzymes toward production of multifarious food from CO2.We report a hybrid electrocatalytic-biocatalytic flow system,coupling photovoltaics-powered electrocatalysis (CO2 to formate)with five enzyme cascade platform(formate to sugar) engineered via genetic mutation. This flow system can in principle be programmed by coupling with diverse enzymes toward production of multifarious food from CO2. For quantitative data, the data points are reported as mean ± standard deviation derived from 3 independent experiments. (n=3)
Research sample	Bi NWs, photovoltaic solar cell and enzymes. The samples were not relevant with sampling study and population.
Sampling strategy	Bi NWs were synthesized by a low-temperature aqueous-chemical method. The enzymes were synthesized and purified from engineered bacteria. The photovoltaic solar cell is purchased from the solar cell company. For quantitative data, the data points are reported as mean ± standard deviation derived from 3 independent experiments. (n = 3). Statistical comparisons were carried out by performing two-sided Student's t-test.
Data collection	SEM, TEM, ICP, ITC, HPLC, photovoltaic measurements, electrochemical measurements, enzymes measurements and photo-enzymatic CO2 conversion test. G.L., Y.Z. and Z.L. designed and performed the experiments. G.W. designed the enzyme cascade reactors.
Timing and spatial scale	The experimental data was obtained during this two years in our lab and the test center in University of Science and Technology of China. Each data was collected when the experiment was finished. The experiments were not relevant with sampling study.
Data exclusions	No data were excluded from the analysis.
Reproducibility	For all solar driven electrocatalytic-biocatalytic CO2 conversion experiments, three independent repeats were performed and the results were reproducible.
Randomization	Not relevant. The samples were prepared and used in each laboratory experiment. The study was not relevant with random grouping or samping studies of animals and clinical trial.
Blinding	The study works with commercial photovoltaic solar cell, prepared Bi NWs and enzymes in lab, and there is no reason for blinding.

Did the study involve field work? ☐ Yes ☒ No

Reporting for specific materials, systems and methods

We require information from authors about some types of materials, experimental systems and methods used in many studies. Here, indicate whether each material, system or method listed is relevant to your study. If you are not sure if a list item applies to your research, read the appropriate section before selecting a response.

Materials & experimental systems

n/a	Involved in the study
☒	☐ Antibodies
☒	☐ Eukaryotic cell lines
☒	☐ Palaeontology and archaeology
☒	☐ Animals and other organisms
☒	☐ Clinical data
☒	☐ Dual use research of concern
☒	☐ Plants

Methods

n/a	Involved in the study
☒	☐ ChIP-seq
☒	☐ Flow cytometry
☒	☐ MRI-based neuroimaging
