# Supplementary material for: Solar-driven sugar production directly from CO2 via a customizable electrocatalytic–biocatalytic flow system
Source: Nat Commun. 2024 Mar 25;15:2636. doi: 10.1038/s41467-024-46954-w (PMC10963751; doi:10.1038/s41467-024-46954-w)
Supplement: Supplementary file 1 — Supplementary Information [file 41467_2024_46954_MOESM1_ESM.pdf]

Supplementary information

Solar-driven sugar production directly from CO_2 via a customizable electrocatalytic–biocatalytic flow system

Supplementary Information for

Solar-driven sugar production directly from CO_2 via a customizable electrocatalytic–biocatalytic flow system

Guangyu Liu[1,2], Yuan Zhong[1], Zehua Liu[1], Gang Wang[3,4], Feng Gao[1], Chao Zhang[1], Yujie Wang[1], Hongwei Zhang[1], Jun Ma[1], Yangguang Hu[1], Aobo Chen[1], Jiangyuan Pan[1], Yuanzeng Min[1], Zhiyong Tang[3,4,5], Chao Gao[1]* & Yujie Xiong[1,2,5]*

[1]Hefei National Research Center for Physical Sciences at the Microscale, Collaborative Innovative Center of Chemistry for Energy Materials (iChEM), School of Chemistry and Materials Science, University of Science and Technology of China, Hefei, Anhui 230026, China.

[2]Suzhou Institute for Advanced Research, Nano Science and Technology Institute, University of Science and Technology of China, Suzhou, Jiangsu 215123, China.

[3]CAS Key Laboratory of Low-Carbon Conversion Science and Engineering, Shanghai Advanced Research Institute, Chinese Academy of Sciences, Shanghai, 201203, China.

[4]School of Chemical Engineering, University of Chinese Academy of Sciences, Beijing, 100049, China.

[5]Key Laboratory of Functional Molecular Solids, Ministry of Education, Anhui Engineering Research Center of Carbon Neutrality, College of Chemistry and Materials Science, Anhui Normal University, Wuhu, Anhui 241000, China.

These authors contributed equally: Guangyu Liu, Yuan Zhong, Zehua Liu.

*e-mail: gaoc@ustc.edu.cn; yjxiong@ustc.edu.cn

Supplementary Methods

Materials

Bi nanopowders (NPs) were purchased from Macklin (Shanghai, China). The anion and cation exchange membranes were purchased from Dioxide Materials (Sustainion X37-50 Grade 60, with a thickness of 50 μm) and Dupont (Nafion 117, with a thickness of 183 μm), respectively, and need further treatment before use. The solid-state electrolyte (AmberChrom 1 × 8 chloride form) was purchased from Sigma-Aldrich (USA). The nicotinamide adenine dinucleotide (NAD^+), reduced nicotinamide adenine dinucleotide (NADH), thiamine pyrophosphate (TPP), 3-(N-morpholino)propanesulfonic acid (MOPS), 2-[4-(2-hydroxyethyl)piperazin-1-yl]ethanesulfonic acid (HEPES), triethanolamine (TEA), formaldehyde, dihydroxyacetone, L-glyceraldehyde and L-sorbose were purchased from Aladdin (Shanghai, China). Phosphate-buffered saline (PBS), BCA kit, sodium dodecyl sulfate-polyacrylamide gel electrophoresis (SDS-PAGE) kit, bovine serum albumin, DNase, RNase and protease inhibitor mixture were purchased from Beyotime Biotechnology (Shanghai, China). Genes and primers were synthesized by General biol (Anhui, China). DNA polymerase and restriction enzymes were purchased from Thermo Fisher Scientific Inc (Waltham, MA, USA). pET21a (+) and pET28a (+) were purchased from Novagen (Madison, WI, USA). The plasmid isolation kit, Tris-HCl, glycerol, and Ni-NTA super flow column for purification were purchased from Sangon Biotech (Shanghai, China). Electrophoresis reagents were purchased from Bio-Rad Laboratories (Hercules, CA, USA). All other reagents were purchased from Sinopharm Chemical Reagent Company (Shanghai, China). All reagents were used as received without further purification. The water used in all experiments was purified using a Millipore Milli-Q water system (resistance was 18.2 MΩ cm).

Materials characterization

Transmission electron microscopy (TEM) images were acquired on a Hitachi 7700 transmission electron microscope and JEM-2100F transmission electron microscope. Scanning electron microscopy (SEM) was performed on a Zeiss Supra 40 VP. Powder

X-ray diffraction (XRD) was performed on a Philips X'Pert Pro Super X-ray diffractometer (Netherlands) with Cu Kα radiation (λ=1.5406 Å). X-ray photoelectron spectra (XPS) were collected on a Kratos Axis supra+ X-ray photoelectron spectrometer, using monochromatic Al Kα X-ray (1486.6 eV) as the excitation source.

Treatment of anion and cation exchange membranes

The purchased anionic and cationic membranes were cut to a size of 3 × 3 cm to fit the reactor. The newly purchased anionic membrane (Sustainion X37-50 Grade 60) was soaked in a 1 M KOH solution for 8 hours. Then the membrane was continued to be activated for 24 hours in a new 1 M KOH solution to complete the activation process. After cutting to the appropriate size, the membrane was thoroughly washed with deionized water prior to being loaded in the solid-electrolyte reactor. The activated anionic membrane was stored in 1 M KOH solutions and the membrane was washed with deionized water several times prior to installation in the solid-electrolyte reactor.

The newly purchased cationic membrane (Nafion 117) was first soaked in a 5 wt% H_2O_2 solution at 80°C for 1 hour, followed by soaking in deionized water for 30 minutes. After that, the membrane was soaked in a 5 wt% H_2SO_4 solution at 80°C for 1 hour. Finally, the membrane was soaked in deionized water for 30 minutes to complete the activation process. The activated cationic membrane was stored in deionized water prior to installing in the solid-electrolyte reactor.

Quantification and activity assay of enzymes

The concentration of the proteins was determined by a BCA kit, with bovine serum albumin as a standard protein[1]. The capacity of NADH regeneration for PTDH-WT and PTDH-Mutant was measured in different buffer (PB, HEPES, MOPs and TEA, 100 mM, pH 7.2) at 27 °C. The reaction mixture (10 mM sodium phosphite, 5 mM NAD^+ and enzyme) was incubated at 30 °C for 10 min and subsequently boiled to deactivate the enzyme. The formation of reduced coenzyme was measured by light absorption at 340 nm in a 96-well plate (Corning Costar, NY, USA) at 27 °C. Absorption was

correlated to a molar basis using the extinction coefficient (ε) of 6.22 mM^{-1} cm^{-1} for NAD^+/NADH.

The activity of CcFDH for CO_2-to-formate conversion was measured in different buffers (PB, HEPES, MOPs and TEA, 100 mM, pH 7.2) at 27 °C. As a quantifiable choice, sodium bicarbonate was used as a CO_2 substitute for this experiment. The reaction mixture (50 mM NaHCO3, 5 mM NADH and enzyme) was incubated at 27 °C for 15 min and subsequently boiled to deactivate the enzyme. Formic acid was quantified in the post-reaction liquid using Lang & Lang method[2]. One unit of reduction activity was defined as the amount of enzyme required to produce 1 μmol formate per minute under standard conditions.

The activity of BmfaldDH for formate-to-formaldehyde conversion was measured in different buffers (PB, HEPES, MOPs and TEA, 100 mM, pH 7.2) at 27 °C. The reaction mixture (10 mM HCOONa, 2 mM NADH, and enzyme) was incubated at 27 °C for 15 min and subsequently boiled to deactivate the enzyme. Formaldehyde was quantified in the post-reaction liquid by gas chromatography (GC). One unit of reduction activity was defined as the amount of enzyme required to produce 1 μmol formaldehyde per minute under standard conditions.

The activity of FLS for formaldehyde-to-dihydroxyacetone conversion was measured in different buffers (PB, HEPES, MOPs and TEA, 100 mM, pH 7.2) at 27 °C. The reaction mixture (5 mM formaldehyde, 1 mM $MgSO_4$, 0.1 mM TPP and enzyme) was incubated at 27 °C for 30 min and subsequently boiled to deactivate the enzyme. Dihydroxyacetone was quantified by high-performance liquid chromatography (HPLC). One unit of reduction activity was defined as the amount of enzyme required to produce 1 μmol dihydroxyacetone per minute under standard conditions.

The activity of FSA for glycolaldehyde-to-L-glyceraldehyde conversion was measured in different buffers (PB, HEPES, MOPs and TEA, 100 mM, pH 7.2) at 27 °C. The reaction mixture (5 mM glycolaldehyde and enzyme) was incubated at 27 °C for 30 min and subsequently boiled to deactivate the enzyme. L-glyceraldehyde was quantified by HPLC. One unit of reduction activity was defined as the amount of

enzyme required to produce 1 μmol L-glyceraldehyde per minute under standard conditions.

The activity of FSA^{A129S} for addition of glycolaldehyde and dihydroxyacetone to L-sorbose was measured in different buffers (PB, HEPES, MOPs and TEA, 100 mM, pH 7.2) at 27 °C. The reaction mixture (5 mM glycolaldehyde, 5 mM dihydroxyacetone and enzyme) was incubated at 27 °C for 30 min and boiled to deactivate the enzyme. L-sorbose was quantified by HPLC. One unit of reduction activity was defined as the amount of enzyme required to produce 1 μmol L-sorbose per minute under standard conditions.

Exploration of optimal conditions for enzyme cascade reactions

The effect of temperature on the activity of CcFDH, BmfaldDH, PTDH-WT, PTDH-Mutant, FLS, FSA and FSA^{A129S} was determined by measuring the relative activity of the enzymes over a range of temperatures (20~65°C). Other experimental conditions were the same as previous standard assay protocols. The optimal pH for achieving the maximum activity of recombinant CcFDH, BmfaldDH, PTDH-WT, PTDH-A155N, PTDH-E175A, PTDH-A176R, PTDH-Mutant, FLS, FSA and FSA^{A129S} was determined by incubating the standard assay mixtures in different buffer media. These reactions in sodium citrate (100 mM, pH 3–5), sodium acetate (100 mM, pH 5–7), potassium phosphate (100 mM, pH 7–8) and glycine (100 mM, pH 8–9) buffers. These data were optimized by response surface methodology (RSM). The statistical software package Design-Expert (Stat-Ease Inc., Minneapolis, MN, USA) was used for regression analysis of experimental data and plotting response surfaces. The activity data of CcFDH, BmfaldDH, PTDH-WT, PTDH-A155N, PTDH-E175A, PTDH-A176R, PTDH-Mutant, FLS, FSA and FSA^{A129S} were obtained from triplicate measurements of initial rates at varying concentrations of respective substrates under standard assay conditions. K_m and V_{max} values were determined from nonlinear regression fitting of the Michaelis–Menten equation using Prism 5 (GraphPad Software, Inc, CA, USA). The data used represent the average standard deviation of the statistically relevant data.

Preparation of Bi nanowires

The Bi nanowires (NWs) were prepared using a slightly modified method from a previous report[3]. Typically, 50 mL of deionized water and $BiCl_3$ (2 mmol) was successively added into a beaker under stirring to yield a white precipitate. After 10 min, Zn powder (4 mmol) was added into the above system, and the mixture was continuously stirred at room temperature for 3 h. Then, the excess metal Zn and byproducts were removed using 50 mL of hydrogen chloride aqueous solution (1 M). The resulting black powder was collected by centrifugation and washed with water three times, and finally dried in a vacuum oven at 60 °C overnight.

Analysis of formate during enzyme cascade reactions

Formate was measured by the Lang and Lang method[2]. Samples (100 μL) containing formate were mixed with 200 μL of solution A, 10 μL of solution B, and 700 μL of 100% acetic anhydride, and incubated at 50°C for 1.5-2 h with occasional rapid mixing. A red color developed, which was measured photometrically at 515 nm. Solution A was prepared by dissolving 0.5 g of citric acid and 10 g of acetamide in 100 mL of isopropanol; solution B was prepared by dissolving 30 g of sodium acetate in 100 mL of water. Sodium formate dissolved in 100 mM potassium phosphate buffer (pH 7.2) was used for standard calibration (Supplementary Fig. 50). Furthermore, formate was confirmed and quantified using Liquid 400 MHz NB NMR Spectrometer (Bruker AVANCE AVIII 400). ^1H nuclear magnetic resonance (NMR) spectra were recorded under an argon atmosphere at 298 K and analyzed with water signal (D_2O) suppression using pre-saturation (256 scans).

Analysis of formaldehyde

Formaldehyde was analyzed quantitatively by gas chromatography (Fuli Instruments, China) equipped with a flame ionization detector and a GDX-102 column (30 m × 0.53 mm × 20 μm). The calibration curve was constructed using a standard formaldehyde solution with concentrations ranging from 0 to 20 mM (Supplementary Fig. 50). The samples (1 μL) was injected into the purging and trapping apparatus under the following

conditions: the injector was operated at a constant 200 °C with Ar carrier gas (1.5 mL min^{-1}), the oven temperature was maintained at 40 °C for 1 min and heated to 200 °C by 5 °C min^{-1}, and the detector was run at 250 °C. The formaldehyde concentration was quantified from the peak areas.

Analysis of dihydroxyacetone, L-glyceraldehyde, L-erythrulose and L-sorbose

Dihydroxyacetone, L-glyceraldehyde, L-erythrulose and L-sorbose were measured using an analytical HPLC system (Agilent Technologies 1260 Infinity II) on a Bio-Rad Aminex HPX-87H instrument with a 300 × 7.8 mm column[4]. Samples (20 μL) were injected and eluted under the following conditions: 5 mM H_2SO_4 at a flow rate of 0.5ml min^{-1} under 50 °C, 40 min run time per sample, detection using both refractive index (RI) and ultraviolet (UV) at 192 nm detectors. The amount of product was quantified from the peak areas. The standard curve of dihydroxyacetone, L-glyceraldehyde, L-erythrulose and L-sorbose dissolved in 100 mM TEA buffer (pH 7.2) was used for standard calibration.

Isothermal titration calorimetry (ITC) measurement

Ligand-enzyme interactions were measured by isothermal titration calorimetry (ITC). Titration experiments were performed on a PEAQ-ITC low volume isothermal titration microcalorimeter (MicroCal, Worcestershire County, UK). All solutions were filtered, centrifuged and degassed to avoid bubble formation, and equilibrated to the appropriate temperature prior to each experiment. After cleaning the instrument and syringe using methanol and 14% Decon 90, the syringe was inserted into the reaction cell. Then, stirring (300 rpm) was started and the instrument was equilibrated at 25°C until the baseline was flat and stable. The titration was performed at 25°C with injecting enzyme (BmfaldDH) and 2.0 μL of ligand (sodium formate or NADH) at 120 s intervals for a total of 19 titrations. The titration data were analyzed by using MicroCal PEAQ-ITC analysis software (Malvern Instruments) with an independent model to obtain fit plots and thermodynamic binding data. Each experiment was performed three times, and the data was compared and averaged. The intrinsic molar enthalpy change (ΔH), binding

constant (K_d) and binding stoichiometry (n) of the binding process were obtained from the best fit of the calorimetric data. By using equation of $\Delta G = -R(T)\ln(1/K_d)$, the Gibbs free energy of binding and K_d were calculated from binding affinity measurements, where R is the universal gas constant and T is the temperature in Kelvin. The binding entropy was then estimated using the equation of $\Delta S = (\Delta H - \Delta G)/T$, where ΔH is the average binding enthalpy.

Energy efficiency calculations

The solar-to-fuel conversion efficiency is defined by the ratio of "chemical power out" to "solar power in"[5]. For our experiment, the solar-to-HCOOH conversion efficiency ($EF_{\text{solar to HCOOH}}$) can be defined as:

$$EF_{\text{solar to HCOOH}} = \frac{P_{\text{HCOOH}}}{P_{\text{Solar}}} = \frac{E^0_{\text{HCOOH/CO}_2} \times J_{\text{Solar}} \times FE_{\text{HCOOH}}}{P_{\text{Solar}}}$$

$$= \frac{1.40\ \text{V} \times 3.41 \frac{\text{mA}}{\text{cm}^2} \times 82.2\%}{100 \frac{\text{mW}}{\text{cm}^2}} = 3.9\% \qquad (1)$$

where P_{HCOOH} is the chemical power for the production of HCOOH from CO_2, P_{Solar} is the incident solar power on the solar cell, $E^0_{\text{HCOOH/CO}_2}$ (1.40 V) is the equilibrium potential for the reaction, J_{solar} (3.41 mA cm^{-2}) is the average current density during the reaction, FE_{HCOOH} (82.2%) is the average Faradaic efficiency of HCOOH production, and P_{Solar} is the light intensity (100 mW cm^{-2}).

$$CO_2(g) + H_2O(l) \rightarrow HCOOH(l) + \tfrac{1}{2}O_2(g) \qquad (2)$$

During the enzyme catalysis process, the bio-consumption of 16.824 g/L (~200 mM) formate yielded 3.10 g/L (~17.2 mM) L-sorbose. As the enthalpy of combustion is –269.9 kJ/mol for formic acid and –2799.1 kJ/mol for L-sorbose according to the data from the National Institute of Standards and Technology (NIST), the calculated $EE_{biocatalysis}$ of enzyme catalysis should be:

$$EE_{biocatalysis} = \frac{-2799.1 \frac{\text{kJ}}{\text{mol}} \times 17.2\ \text{mM}}{-269.9 \frac{\text{kJ}}{\text{mol}} \times 200\ \text{mM}} \times 100\% = 89.2\% \qquad (3)$$

Accordingly, the overall energy efficiency was calculated as:

$$EE(\text{total}) = EE(\text{Solar to HCOOH efficiency}) \times EE(\text{biocatalysis})$$
$$= 3.9\% \times 89.2\ \% = 3.5\% \quad (4)$$

Supplementary Figures

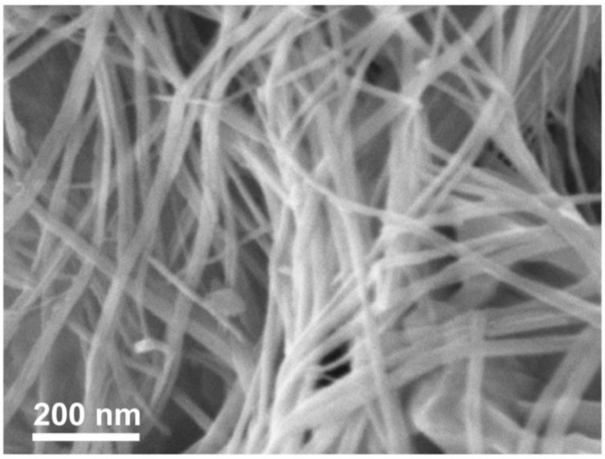

Supplementary Fig. 1 | SEM image of prepared Bi NWs.

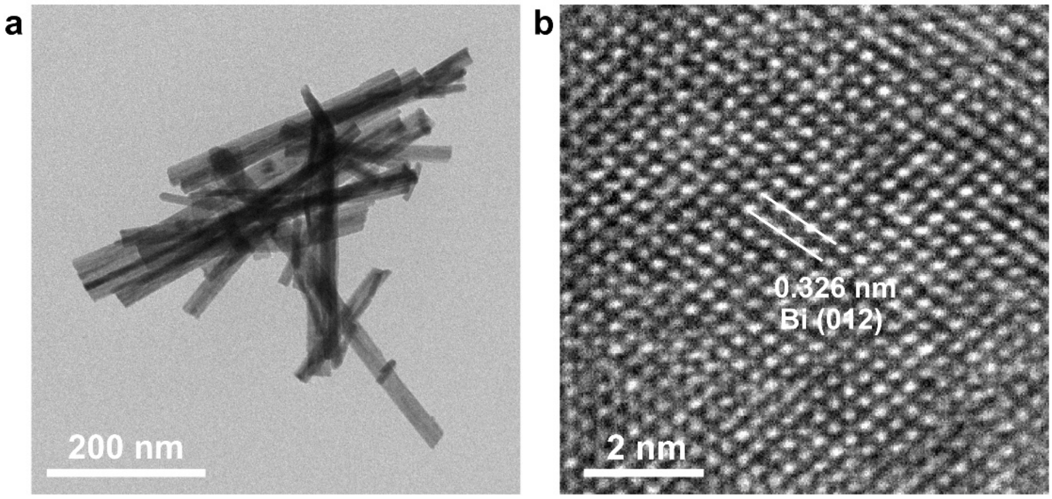

Supplementary Fig. 2 | (**a**) TEM image and (**b**) high-resolution TEM image of prepared Bi NWs.

The high-resolution TEM image of prepared Bi NWs shows a fringe spacing of 0.326 nm, corresponding to the (012) facet of metallic Bi.

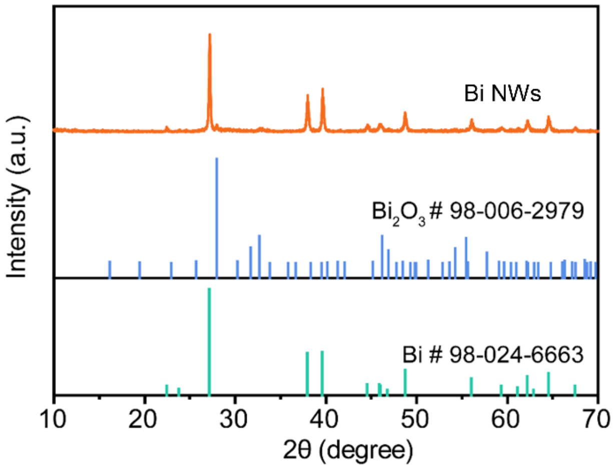

Supplementary Fig. 3 | XRD pattern of prepared Bi NWs. The standard diffraction patterns for anatase Bi_2O_3 (JCPDS No. 98-006-2979) and Bi (JCPDS No. 98-024-6663) are provided as references.

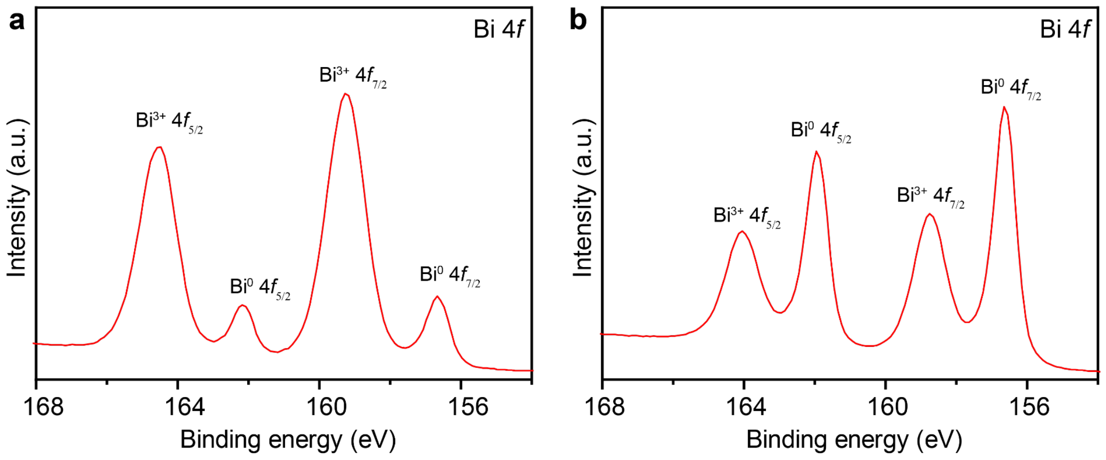

Supplementary Fig. 4 | Bi 4*f* XPS spectra of prepared Bi NWs before (a) and after (b) Ar ion etching.

The Bi 4*f* XPS spectra of prepared Bi NWs (Supplementary Fig. 4a) could be divided into two groups according to binding energies: the two peaks at 158.9 and 164.2 eV are assigned to Bi^{3+} in Bi_2O_3, while the other two peaks at 156.9 and 162.4 eV are assigned to metallic Bi^0. The strong peak signal for Bi^{3+} and relatively weak peak signal of Bi^0 can be attributed to air oxidation of metallic Bi on the surface of Bi NWs. After Ar ion etching, the Bi 4*f* XPS spectra shows a much stronger signal of Bi^0 than that of Bi^{3+} (Supplementary Fig. 4b), confirming the inner metallic character of the prepared Bi NWs.

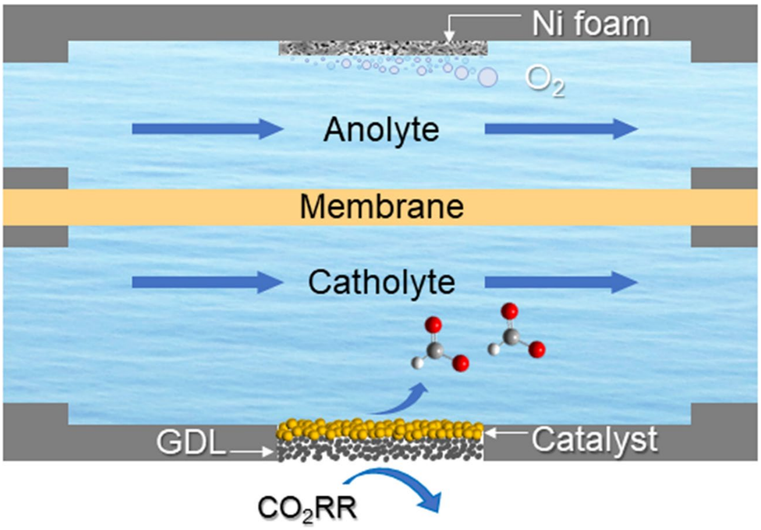

Supplementary Fig. 5 | Schematic illustration of CO_2 reduction to formate in a flow cell with a gas diffusion electrode. GDL, gas diffusion layer.

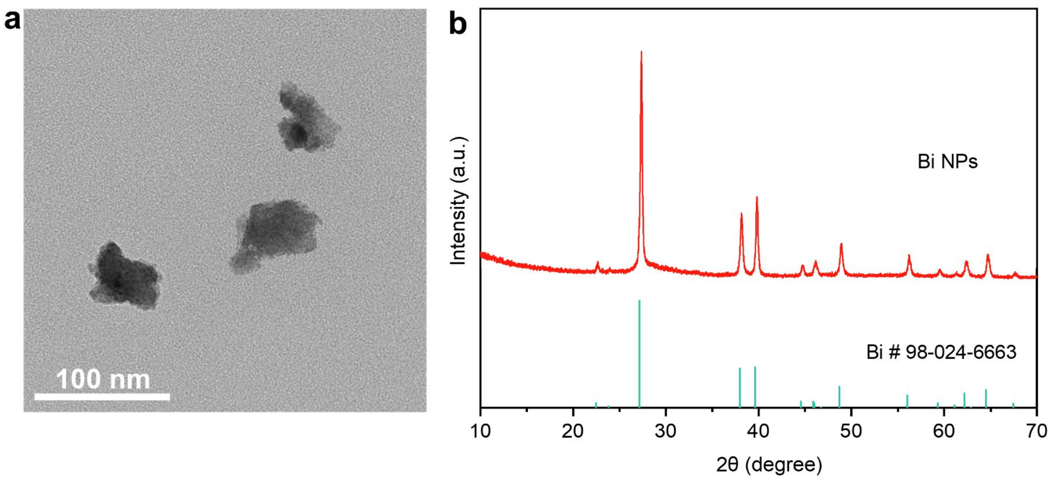

Supplementary Fig. 6 | (**a**) TEM image and (**b**) XRD pattern of the commercial Bi nanopowders (NPs). The standard diffraction patterns for Bi (JCPDS No. 98-024-6663) are provided as references.

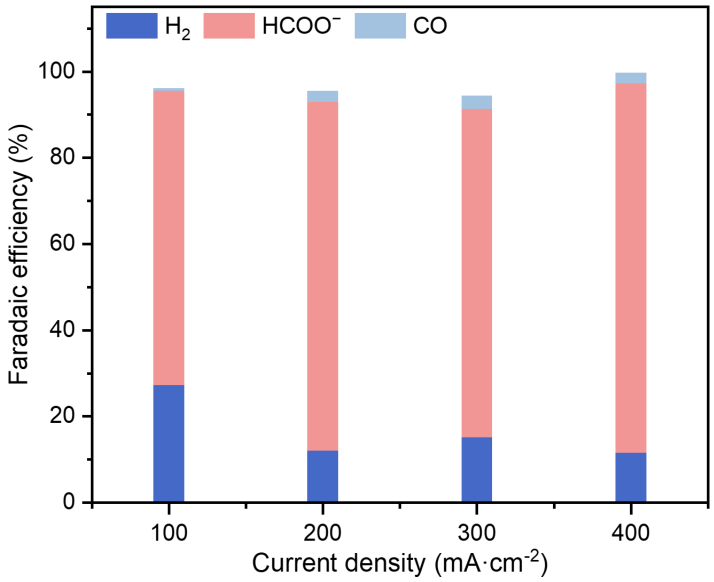

Supplementary Fig. 7 | Faradaic efficiencies of products over commercial Bi NPs catalysts for electrocatalytic CO_2 conversion at different current densities.

The Bi NWs catalyst shows higher activity and selectivity for CO_2-to-HCOOH conversion in contrast to Bi NPs, which should be attributed to the higher edge-to-facet ratio of Bi NWs that is conducive to the stabilization of key intermediates for HCOOH production[6].

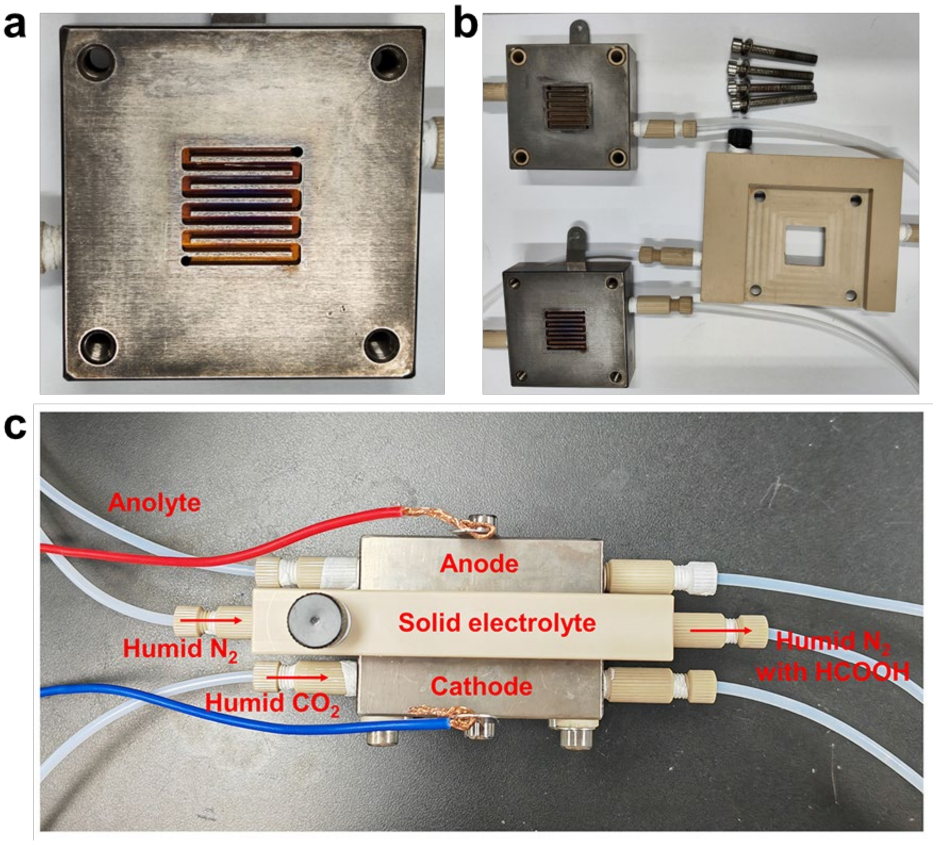

Supplementary Fig. 8 | The digital image of the flow passage (a) and the solid-electrolyte reactor (b, c).

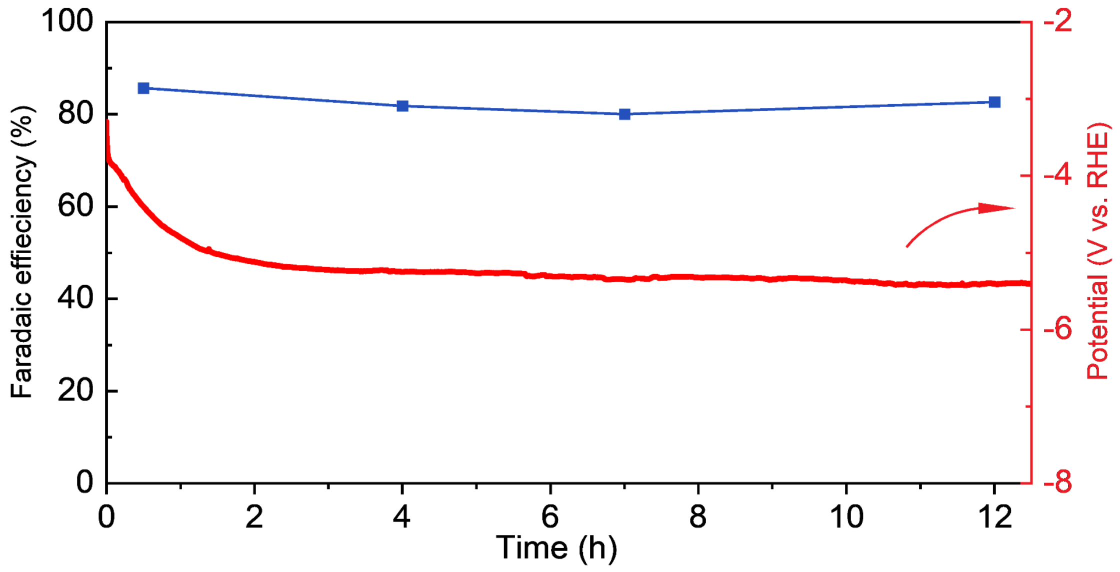

Supplementary Fig. 9 | Measurement of CO_2 reduction to pure HCOOH solution for the prepared Bi NWs catalyst in a solid-electrolyte reactor powered by the electrochemical workstation under the current of 400 mA.

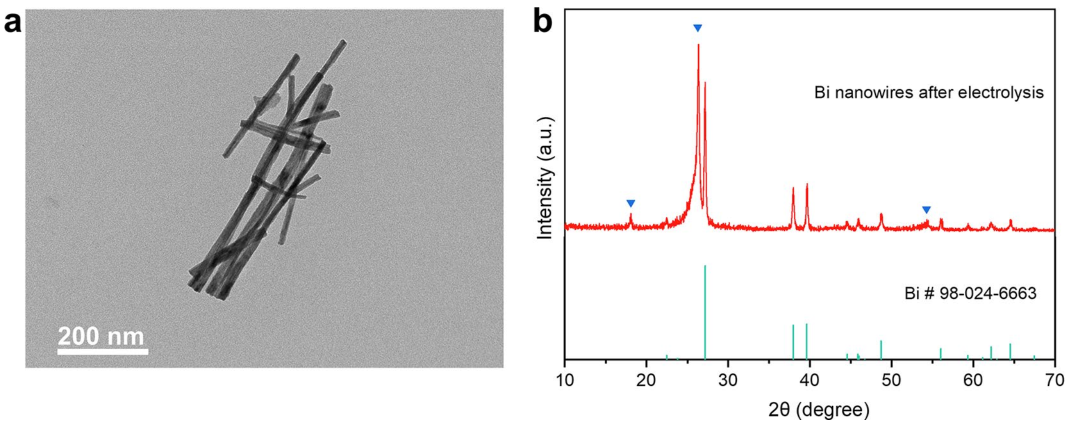

Supplementary Fig. 10 | (a) TEM image and **(b)** XRD pattern of the Bi NWs after electrolysis in a porous solid-electrolyte reactor. The standard diffraction pattern for Bi (JCPDS No. 98-024-6663) is provided as a reference. The XRD profiles of the blank gas diffusion electrode (GDE) are marked with inverted triangles.

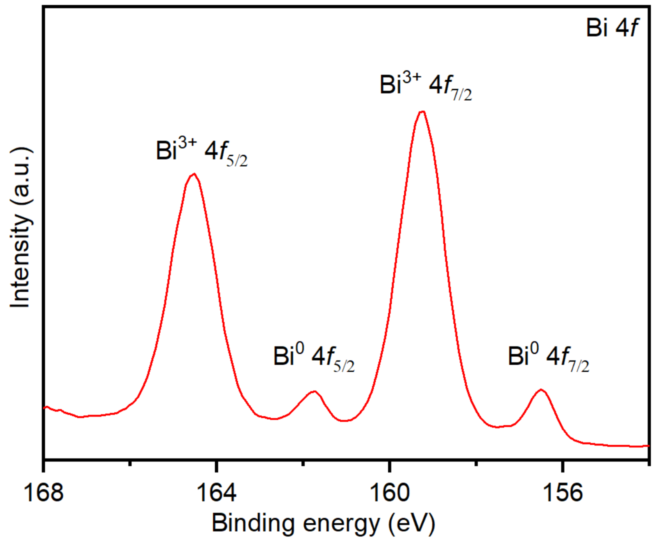

Supplementary Fig. 11 | Bi 4*f* XPS spectra of Bi NWs after 120 h stability test in a porous solid-electrolyte reactor.

The strong peak for Bi^{3+} could be ascribed to the air oxidation of metallic Bi on the surface of Bi NWs after removing the negative potentials for CO_2 reduction reaction.

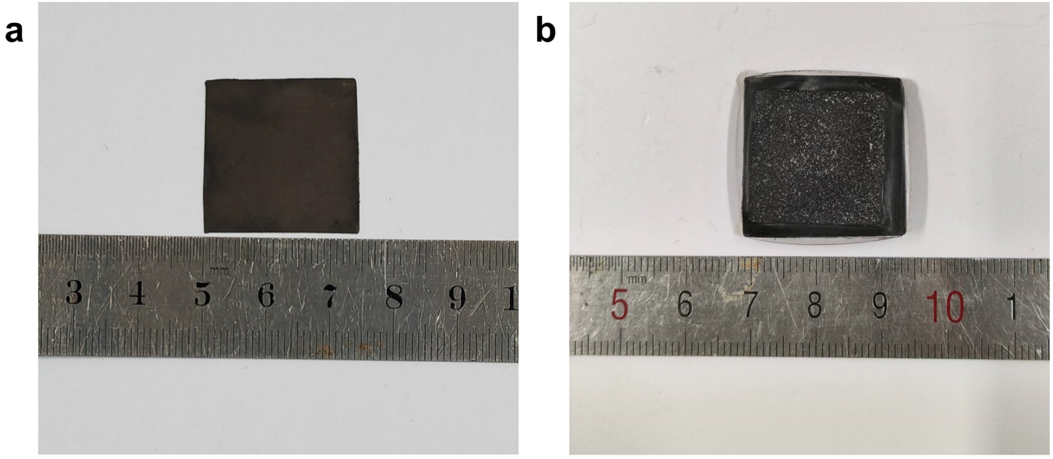

Supplementary Fig. 12 | Photograph of the Bi NWs catalysts coated on GDL. (**a**) before reaction; (**b**) after 120 h stability test (sticked with an anion-exchange membrane).

检 测 报 告
Test Report

报告编号：23Q3-00091
Report No.

客户名称 Name of Customer	University of Science and Technology of China(USTC)
联络信息 Contact Information	96 Jinzhai Road, Hefei, Anhui Province, P.R. China
物品名称 Name of items	Solar cell
型号/规格 Type /Specification	/
物品编号 Items No	USTC20230327
制造厂商 Manufacturer	/
物品接收日期 Items Receipt Date	2023-03-27
检测日期 Test Date	2023-03-30

（盖章处）
Stamp

批准人 Approved by 黎健生 黎健生
核验员 Checked by 何翔 何翔
检测员 Test by 江忠原 江忠原

发布日期 Date of Report 2023 年 Year 04 月 month 03 日 Day

扫一扫 查真伪

本院/本中心地址：福州市屏东路 9-3 号
Address : 9-3 Pingdong Road,Fuzhou,China
电话：0591-87845050
Telephone
传真：0591-87808417
Fax
邮编：350003
Post Code
网址：www.fjjl.net
Web Site
咨询电话：0591-87845050
Inquire line
投诉电话：0591-87823025
Complaint Tel

未经本院/本中心书面批准，部分复制采用本报告内容无效。
Partly using this Report will not be admitted unless allowed by FMI/ Center.

检测结果/说明：
Results of Test and Additional Explanation.

1 Standard Test Condition (STC)：Total Irradiance：1000 W/m^2
Temperature：25.0 ℃
Spectral Distribution：AM1.5G

2 Measurement Data and I-V/P-V Curves under STC

	I_{sc}（A）	V_{oc} (V)	*FF* (%)	η（%）	P_{MPP} (W)	I_{MPP} (A)	V_{MPP} (V)
1	0.1505	5.843	68.72	15.53	0.6043	0.1340	4.510
2	0.1506	5.852	68.40	15.50	0.6028	0.1320	4.567
3	0.1505	5.840	68.61	15.50	0.6030	0.1340	4.500
average	0.1505	5.845	68.58	15.51	0.6034	0.1333	4.526

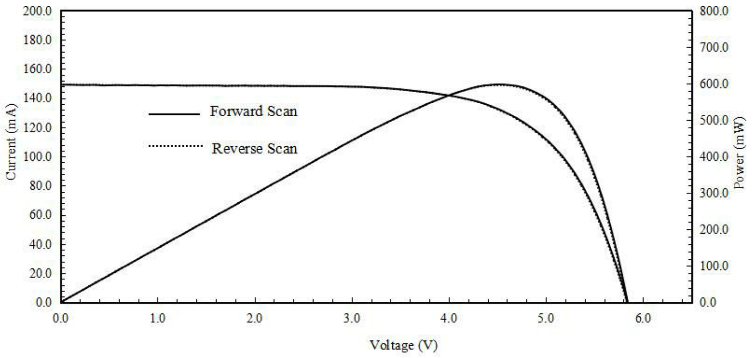

Figure 1. I-V and P-V characteristic curves of the measured sample under STC

3 Pictures of the Measured Sample

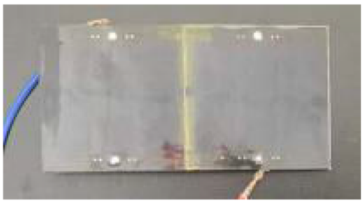

Figure 2. appearance of the sample

检测结果/说明：
Results of Test and Additional Explanation.

Uncertainty of Measurement Results:

Short-Circuit Current: U_{rel}=1.8% (k=2); Open-Circuit Voltage: U_{rel}=1.0% (k=2);

Maximum Power: U_{rel}=2.2% (k=2); Efficiency: U_{rel}=2.2% (k=2); Fill Factor: U_{rel}=3.2% (k=2).

说明：The designated illumination area of the measured sample was 38.9 cm^2.
Explanation

Testing Method (Code and Name) for This Test
IEC 60904-1: 2020 Photovoltaic devices- Part 1: Measurement of photovoltaic current-voltage characteristics

Measurement Standards Used in This Test					
**Name**	**Number**	**Measuring Range**	**Uncertainty or Accuracy Class or Maximum Permissible Error**	**Name of Traceability Institution/Certificate No.**	**Due Date**
SourceMeter	10807C00878-2	DCI：(-10μA~1A)；DCV：(20mV~20V)	Measure：DCV:U_{rel}=0.05%,k=2; DCI：U_{rel}=0.05%,k=2 Output：DCV:U_{rel}=0.05%,k=2; DCI：U_{rel}=0.05%,k=2	Fujian Metrology Institute/ 22D2-01826	2023-04-13
Solar Simulator	2015-006	(300～1200) nm；(800～1200) W/m^2	Spectral Match：(300～360) nm:U_{rel}=8.0% (k=2);(360～1200) nm:U_{rel}=6.2% (k=2);Irradiance Ratio: U_{rel}=1.2% (k=2)	Fujian Metrology Institute/ 22Q2-00720	2023-06-16
WPVS Monocrystalline Silicon Reference Cell	015-2014	(300～1200) nm	U_{rel}=1.3% (k=2)	National Institute of Metrology/ GXgf2021-10725	2023-04-05
Digital Thermometer	15-B	(15～65) ℃	U=0.1 ℃ (k=2)	Fujian Metrology Institute/22B2-07588	2023-06-20

以下空白
Blank below

Supplementary Fig. 13 | The third-party certified test report for the commercial Si photovoltaic cell that was used for powering the electrocatalysis module. The effective surface size of the photovoltaic cell is 96.0 mm × 40.5 mm.

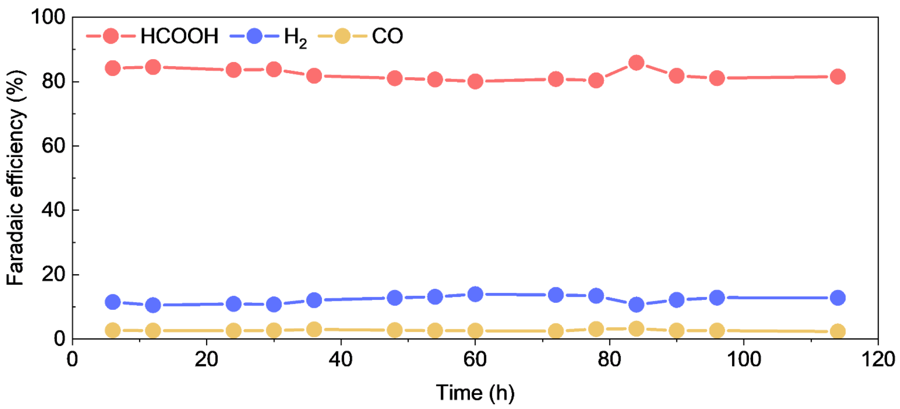

Supplementary Fig. 14 | Faradaic efficiencies for the related products during the 120 h of solar-driven one-step electrocatalytic CO_2 conversion to formic acid.

The full-cell energy loss of the system was calculated as follows[7,8]:

$$\text{Energy loss} = [1 - (1.23 - E_{HCOOH}) \times FE_{HCOOH}/E_{cell})] \times 100\%$$

where E_{HCOOH} is the thermodynamic potential (versus RHE) for CO_2 reduction to HCOOH, which is -0.17 V[9]. FE_{HCOOH} is the Faradaic efficiency for HCOOH, which is 82.2% by taking the average value during the 120 h solar-driven electrocatalytic reaction. E_{cell} is the cell voltage of the solid-electrolyte reactor, which is 4.53 V by taking the average value during the 120 h solar-driven electrocatalytic reaction. As such, the full-cell energy loss of our PV-EC system was calculated to be 74.6%. We would like to point out that the full-cell energy conversion efficiencies of the solid-electrolyte reactor are commonly relatively low at high cell voltage[7,10]. The energy loss is ascribed to the high cell voltage that we used to enable the operating point of the EC close to the maximum power point of the PV, as well as the high overpotential of water oxidation and the internal resistance of the electrolyzer.

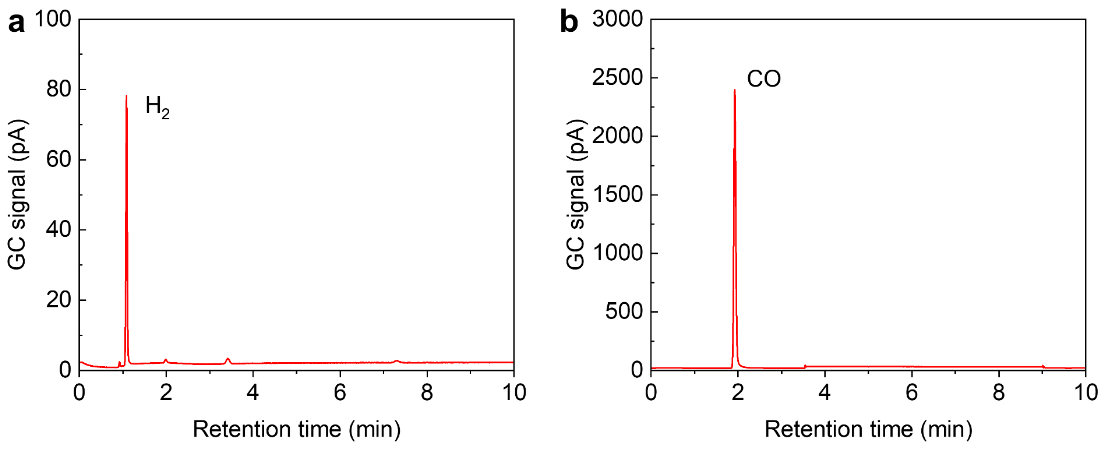

Supplementary Fig. 15 | The gas chromatogram for analysis of the evolved gaseous products during electrocatalytic CO_2 reduction. **a**, CO was detected by a flame ionization detector. **b**, H_2 was detected by a thermal conductivity detector.

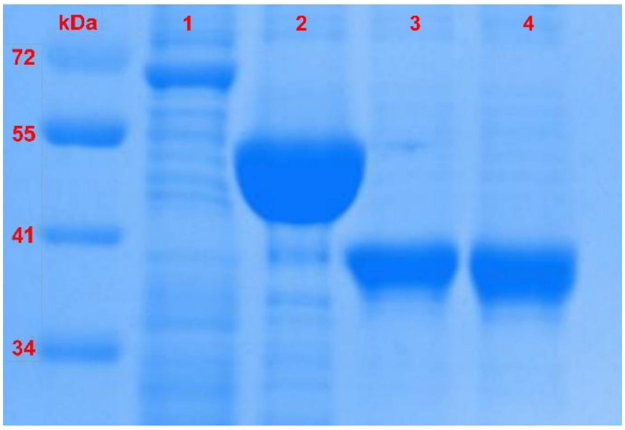

Supplementary Fig. 16 | SDS-PAGE analysis of purified CcFDH (62.9 kDa), BmfaldDH (43.3 kDa), PTDH-WT (36.4 kDa) and PTDH-Mutant (36.4 kDa). 1: purified CcFDH, 2: purified BmfaldDH, 3: purified PTDH-WT, and 4: purified PTDH-Mutant against molecular mass marker.

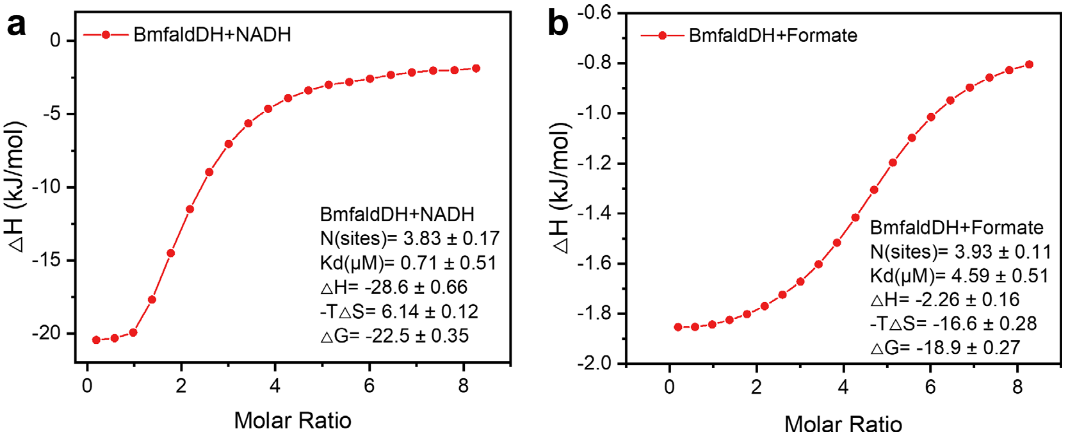

Supplementary Fig. 17 | The binding isotherms analyzed by MicroCal PEAQ-ITC analysis software using independent model. **a**, The enthalpy change per injection for BmfaldDH and formate. **b**, The enthalpy change per injection for BmfaldDH and NADH.

The negative ΔG value for both the binding of BmfaldDH to formate and NADH indicate that BmfaldDH is thermodynamically capable of catalyzing the formate-to-formaldehyde conversion, and this formate reduction conversion is favored when formate and NADH are present in excess in the reaction mixture.

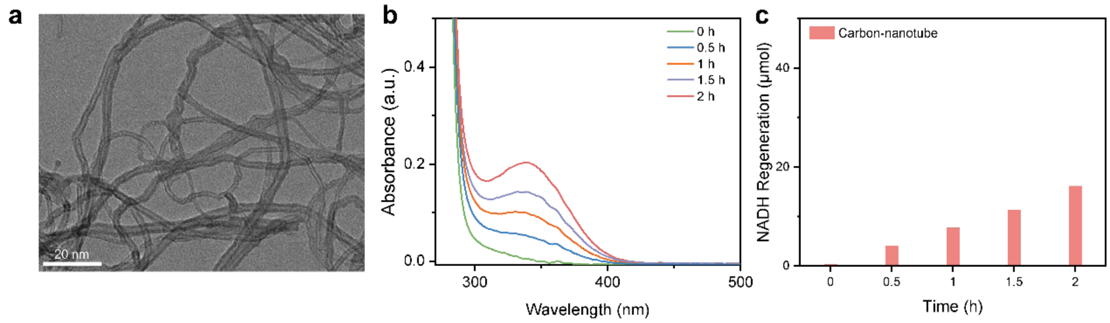

Supplementary Fig. 18 | a, SEM image of commercial carbon nanotubes for electrocatalytic NADH regeneration. **b**, UV-vis absorption spectra of NADH regeneration using electrocatalytic carbon nanotubes (absorption at 340 nm represents NADH). **c**, The amount of regenerated NADH by electrocatalysis over commercial carbon nanotubes (the absorption coefficient of NADH at 340 nm is 6.22 mM^{-1} cm^{-1}).

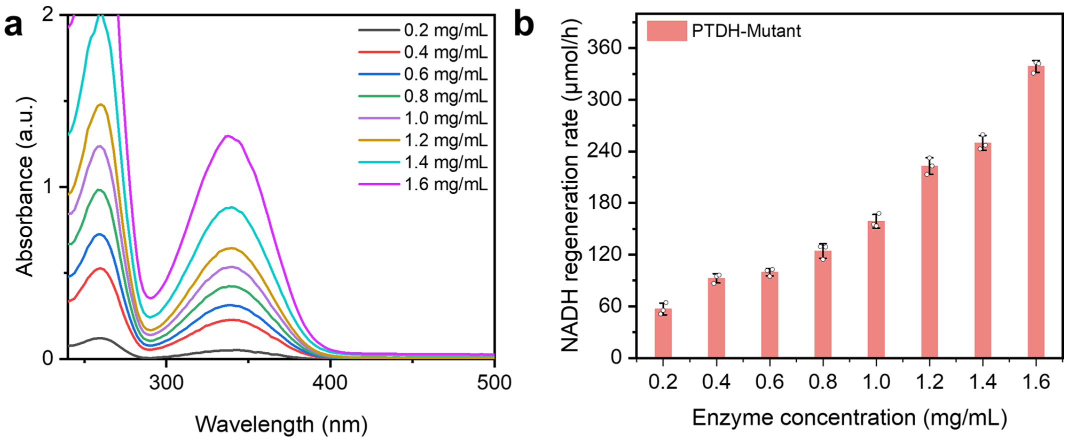

Supplementary Fig. 19 | a, UV-vis absorption spectra of PTDH-Mutant with different concentrations for NADH regeneration (absorption at 340 nm represents NADH). **b**, The amount of regenerated NADH over PTDH-Mutant with different concentrations (the absorption coefficient of NADH at 340 nm is 6.22 mM^{-1} cm^{-1}). Data points are reported as mean ± standard deviation derived from 3 independent experiments (n = 3).

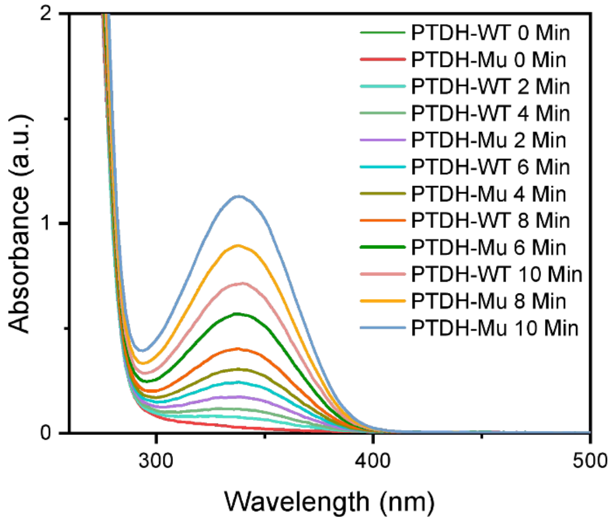

Supplementary Fig. 20 | UV-vis absorption spectra of PTDH-WT and PTDH-Mutant (PTDH-Mu) for NADH regeneration with different reaction time (absorption at 340 nm represents NADH).

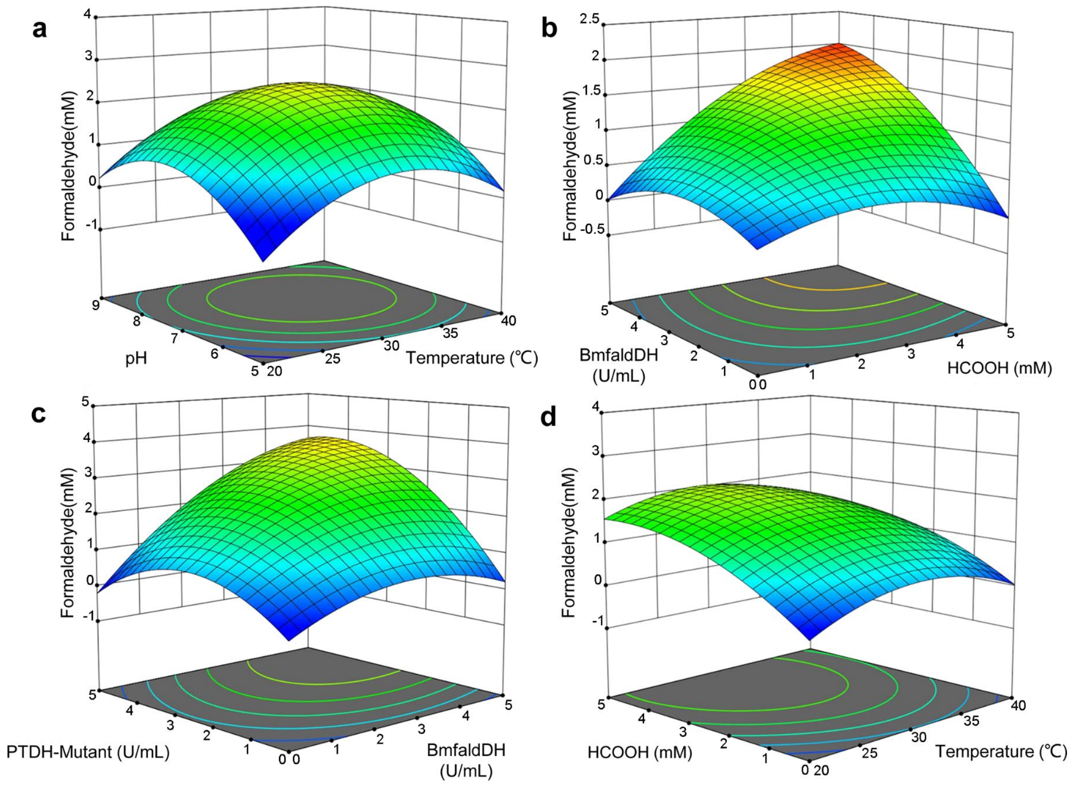

Supplementary Fig. 21 | Response surface plots showing the interaction effect of variables on formaldehyde yeild in formate-to-formaldehyde conversion. **a**, pH versus temperature; **b**, BmfaldDH concertation versus formate concertation; **c**, PTDH-Mutant concertation versus BmfaldDH concertation; **d**, temperature versus formate concertation.

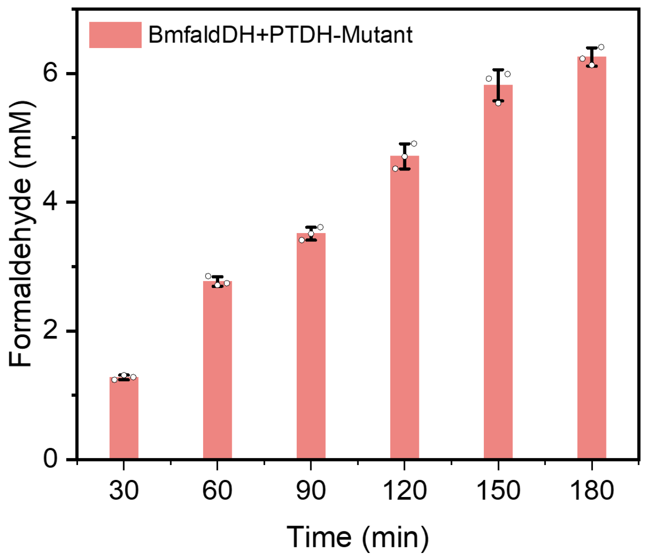

Supplementary Fig. 22 | The yield of formaldehyde produced by BmfaldDH–PTDH-Mutant enzymatic system. Reaction conditions: 10 mM sodium formate, 2 mM NAD^+ and 50 mM sodium phosphite in TEA buffer. The concentrations of BmfaldDH and PTDH-Mutant were 2 mg/mL. Data points are reported as mean ± standard deviation derived from 3 independent experiments (n = 3).

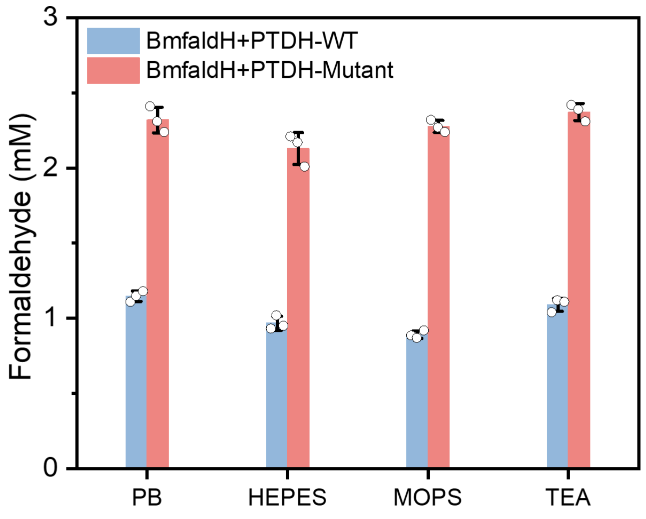

Supplementary Fig. 23 | The yield of formaldehyde produced by BmfaldDH–PTDH-Mutant and BmfaldDH–PTDH-WT enzymatic systems in different buffers (PB, HEPES, MOPS and TEA). Reaction conditions: 5 mM sodium formate, 1 mM NAD^+ and 30 mM sodium phosphite. The concentrations of BmfaldDH, PTDH-WT and PTDH-Mutant were 2 mg/mL. Data points are reported as mean ± standard deviation derived from 3 independent experiments (n = 3).

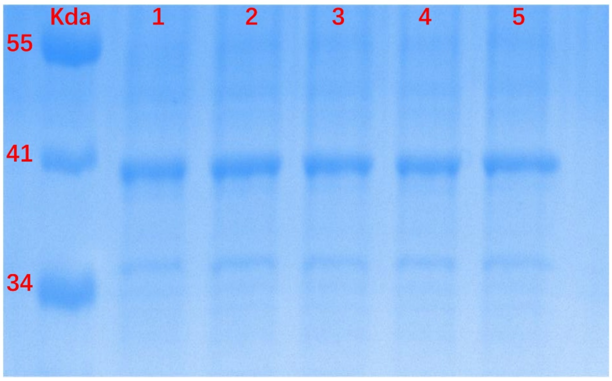

Supplementary Fig. 24 | SDS-PAGE analysis of purified PTDH-WT, PTDH-A155N, PTDH-E175A, PTDH-A176R and PTDH-Mutant (all 36.4 kDa). 1: purified PTDH-WT, 2: purified PTDH-A155N, 3: purified PTDH-E175A, 4: purified A176R, and 5: purified PTDH-Mutant against molecular mass marker.

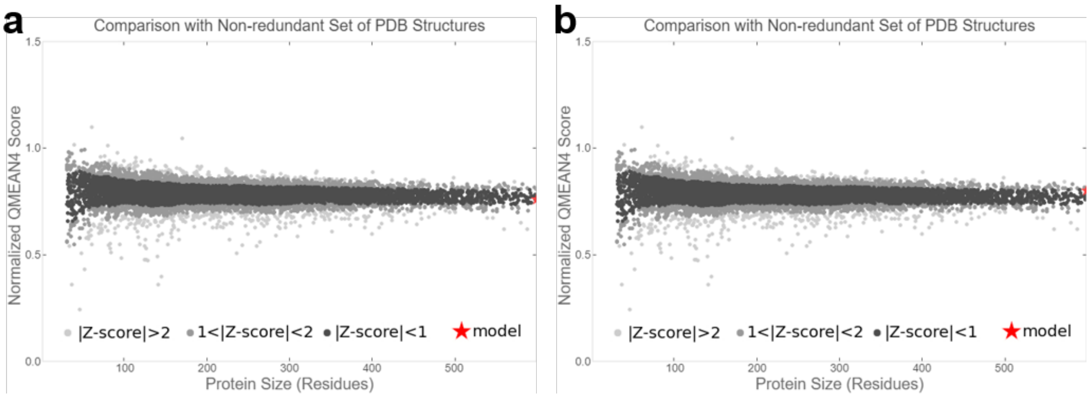

Supplementary Fig. 25 | The qualitative model analysis of (**a**) PTDH and (**b**) PTDH-Mutant.

The QMEAN scores of PTDH and PTDH-Mutant were 0.9±0.05 and 0.90±0.03, respectively. The GMQE scores of PTDH and PTDH-Mutant were 0.95 and 0.94, respectively. The results show high confidence based on homology modeling.

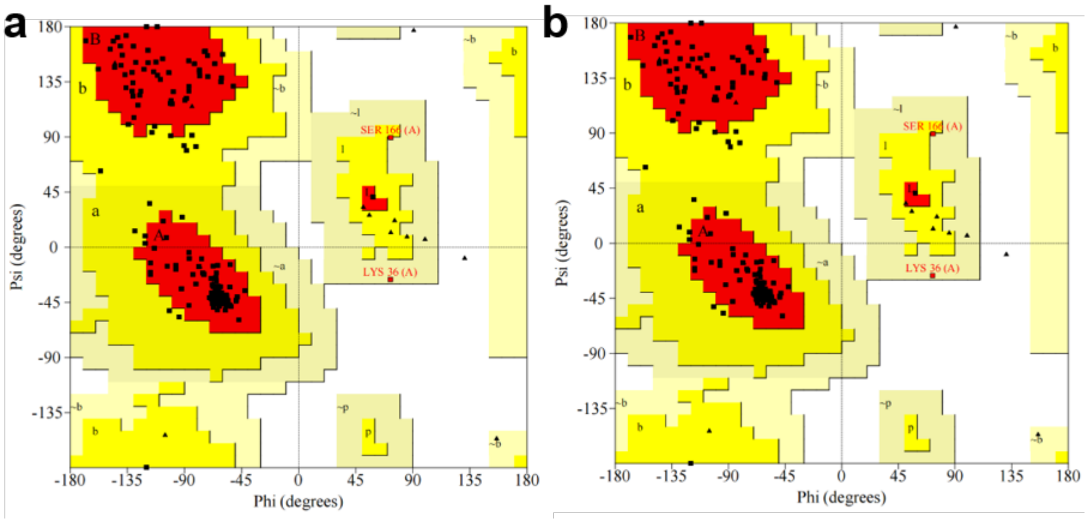

Supplementary Fig. 26 | The Ramachandran plot of homology modeling with (**a**) PTDH and (**b**) PTDH-Mutant.

The proportion of amino acid residues falling in the permissive and maximal permissive regions is higher than 90% of the whole protein, indicating that the conformations of PTDH and PTDH-Mutant models conform to the rules of stereochemistry. The results show the high confidence and accuracy of this homology modeling.

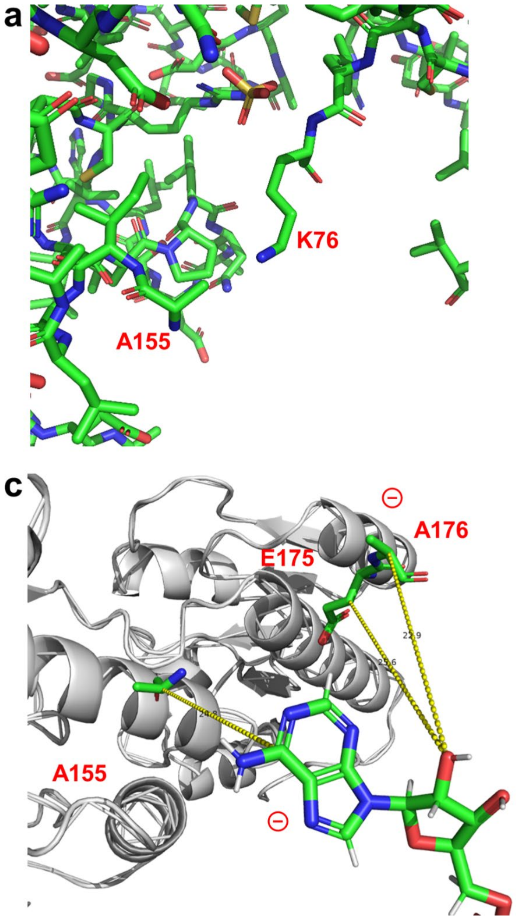

Supplementary Fig. 27 | Homology modeling of (**a**) PTDH-WT and (**b**) PTDH-A155N with K76 interaction. Homology modeling of (**c**) PTDH-WT and (**d**) PTDH-A155N, E175A and A176R with NAD^+ (partial) interaction.

When PTDH contacts with NAD^+, K76 in PTDH is one key amino acid for binding with NAD^+, by forming hydrogen bonds and salt bridges with phosphate groups in NAD^+. The homologous modeling shows that A155N mutation can extend over the NAD^+ phosphate and form the hydrogen bond with K76[11], strengthening the existing salt bridge between K76 and phosphate group in NAD^+. This interaction may promote the pre-organization of K76 to capture NAD^+ during binding process and enhance the binding affinity of NAD^+ to PTDH-A155N (Supplementary Fig. 27a, b), which is verified by the reduced K_m value of PTDH-A155N in contrast to PTDH-WT. In addition, the two specific residues Glu175 and Ala176 in PTDH also are recognized to hold the

key to determining the binding affinity toward NAD^+ [12]. In this model, the Glu175 residues can form hydrogen bonds with the hydroxyl groups of NAD^+ adenine ribose because their proximity is only 2.8 Å (Supplementary Fig. 27c)[13]. However, electronegative Glu175 will spatially and electrostatically repel the 2'-phosphate of NAD^+, resulting in weak binding with PDTH-WT enzymes. The E175A mutation can replace Glu175 (alanine, glycine, and valine) with uncharged amino acid residues having small spaces[14], thus enhancing the binding affinity toward NAD^+. Additionally, the A176R mutation replaces electronegative Ala176 with an electropositive amino acid, which can interact with the 2'-phosphate of NAD^+ statically and simultaneously bind to the E175A mutant base by hydrogen bonding. This mutation stabilizes the additional negative charge of NAD^+ (Supplementary Fig. 27c, d), further enhances the capacity of PTDH to bind NADH, and thus finally improves the conversion efficiency[15]. These impacts of the two mutations are supported by the kinetic parameters of PTDH-E175A and PTDH-A176R. The K_m values of E175A and A176R mutants are both smaller than that of PTDH-WT, while the k_{cat} values of the E175A and A176R mutants increase significantly in contrast to PTDH-WT, indicating that these two site mutations are beneficial for the regeneration of NADH.

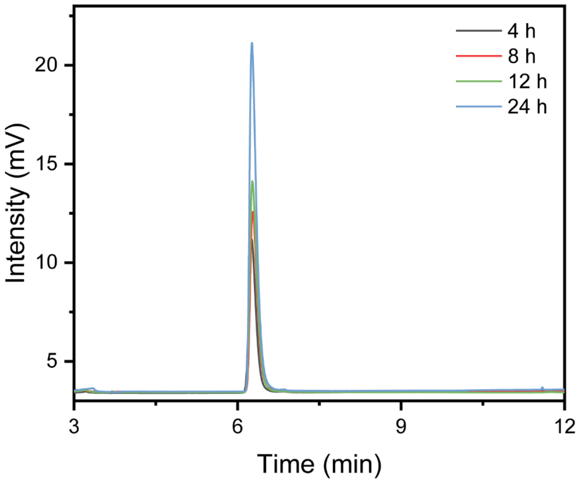

Supplementary Fig. 28 | The gas chromatogram for analysis of produced HCHO in BmfaldDH−PTDH-Mutant enzymatic system.

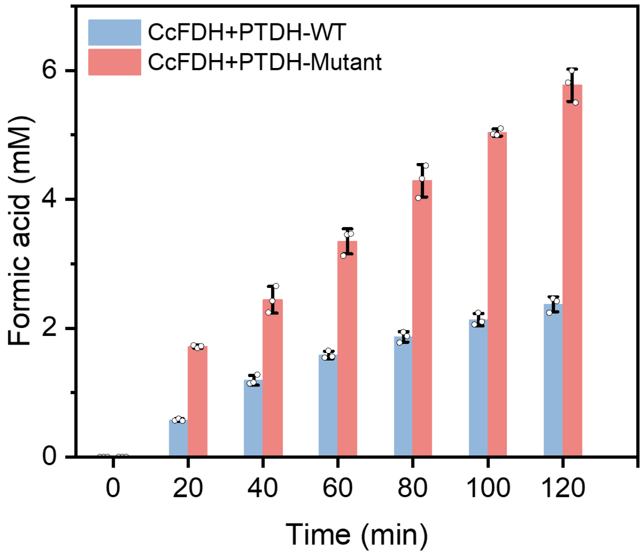

Supplementary Fig. 29 | The yield of formic acid produced by CcFDH–PTDH-Mutant and CcFDH–PTDH-WT enzymatic systems. Reaction conditions: 10 mM sodium bicarbonate, 2 mM NAD^+ and 50 mM sodium phosphite in PBS buffer. The concentrations of CcFDH, PTDH-WT and PTDH-Mutant were 2 mg/mL. Data points are reported as mean ± standard deviation derived from 3 independent experiments (n = 3).

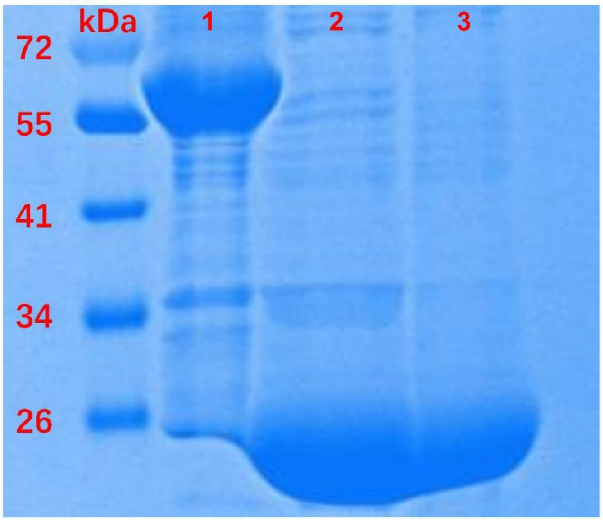

Supplementary Fig. 30 | SDS-PAGE analysis of purified FLS (61.4 kDa), FSA (23 kDa) and FSA^{A129S} (23 kDa). 1: purified FLS, 2: purified FSA, and 3: purified FSA^{A129S} against molecular mass marker.

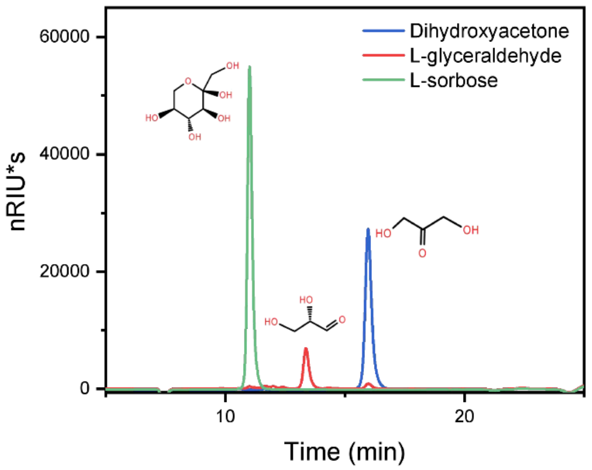

Supplementary Fig. 31 | The HPLC chromatogram of the standards: DHA, L-glyceraldehyde and L-sorbose at 8.5 mM (RI detector 192 nm).

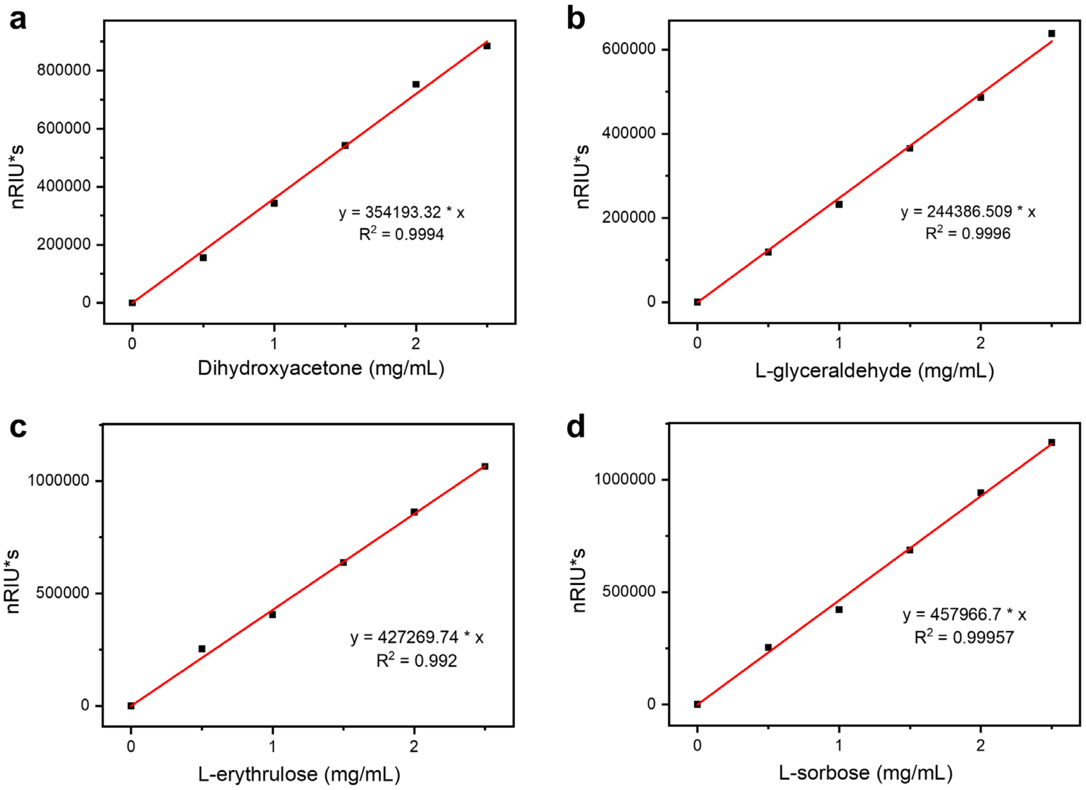

Supplementary Fig. 32 | Calibration curve for quantification of (**a**) dihydroxyacetone, (**b**) L-glyceraldehyde, (**c**) L-erythrulose and (**d**) L-sorbose by HPLC.

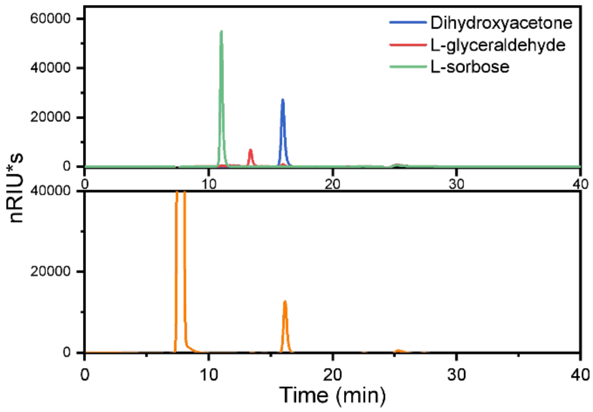

Supplementary Fig. 33 | HPLC analysis of FLS-catalyzed synthesis of dihydroxyacetone from formaldehyde.

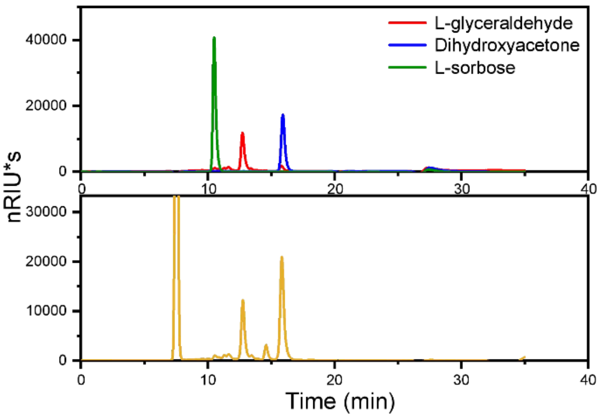

Supplementary Fig. 34 | HPLC analysis of FLS- and FSA-catalyzed synthesis of dihydroxyacetone and L-glyceraldehyde from formaldehyde.

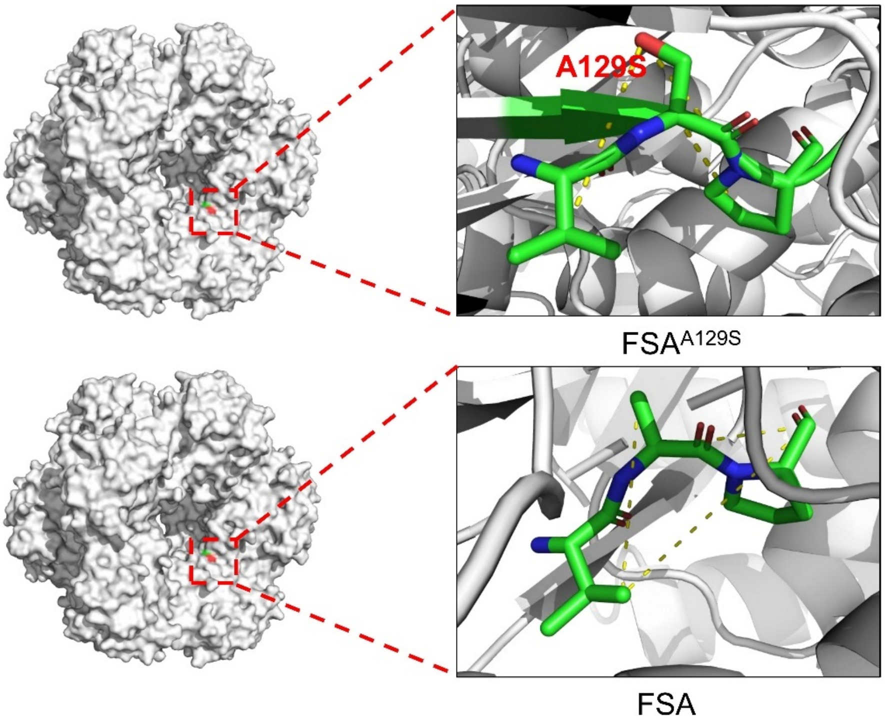

Supplementary Fig. 35 | Homology modeling of FSA and FSA^{A129S} mutant with amplification of amino acids at A129S.

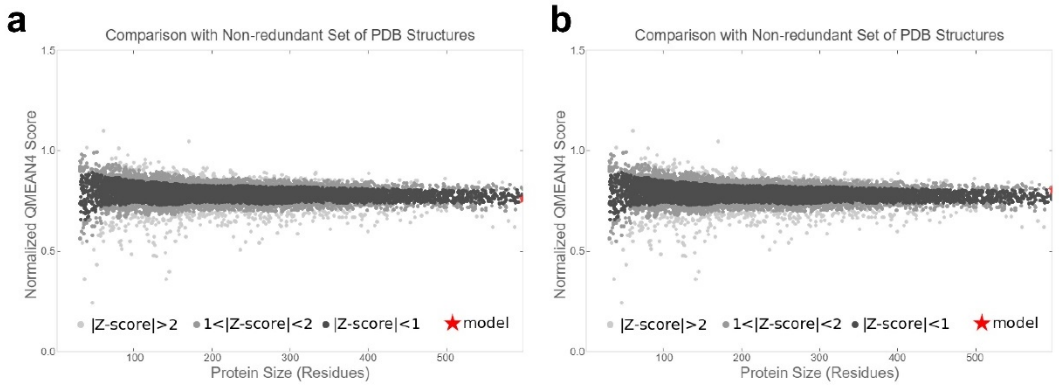

Supplementary Fig. 36 | The qualitative model analysis of (**a**) FSA and (**b**) FSA^{A129S}.

The QMEAN scores of FSA and FSA^{A129S} were 0.91±0.05 and 0.90±0.04, respectively. The GMQE scores of FSA and FSA^{A129S} were 0.97 and 0.98, respectively. The results show high confidence based on homology modeling.

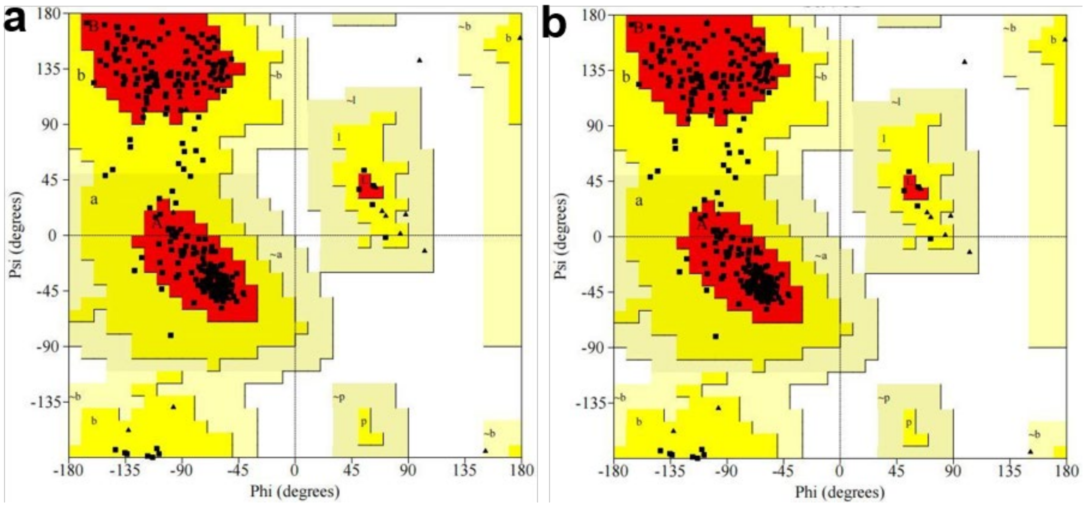

Supplementary Fig. 37 | The Ramachandran plot of homology modeling with (**a**) FSA and (b) FSA^{A129S}.

The proportion of amino acid residues falling in the permissive and maximal permissive regions is higher than 90% of the whole protein, indicating that the conformations of FSA and FSA^{A129S} models conform to the rules of stereochemistry. The results show the high confidence and accuracy of this homology modeling.

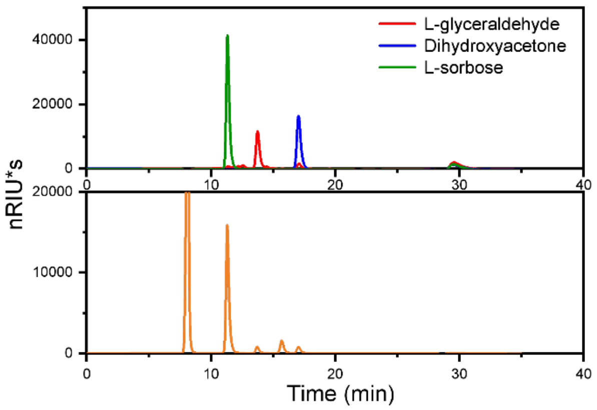

Supplementary Fig. 38 | HPLC analysis of FSA^{A129S}-catalyzed synthesis of L-sorbose from dihydroxyacetone and L-glyceraldehyde.

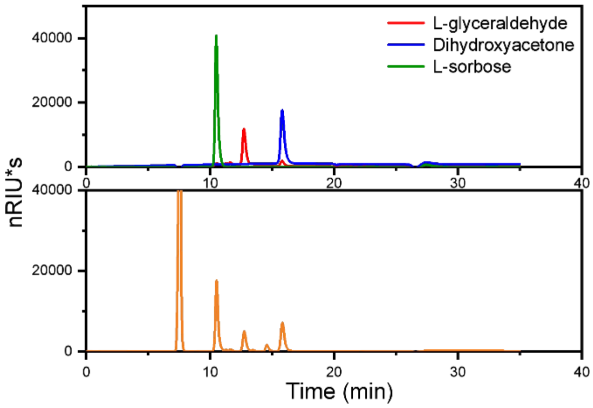

Supplementary Fig. 39 | HPLC analysis of FLS-, FSA- and FSA^{A129S}-catalyzed synthesis of L-sorbose from formaldehyde.

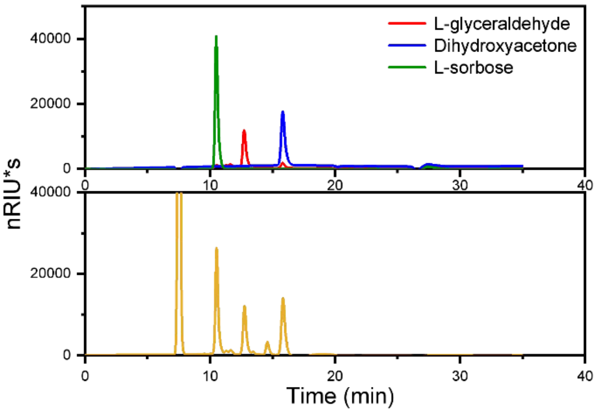

Supplementary Fig. 40 | HPLC analysis of BmfaldDH-, PTDH-Mutant-, FLS-, FSA- and FSA^{A129}-catalyzed synthesis of L-sorbose from formaldehyde.

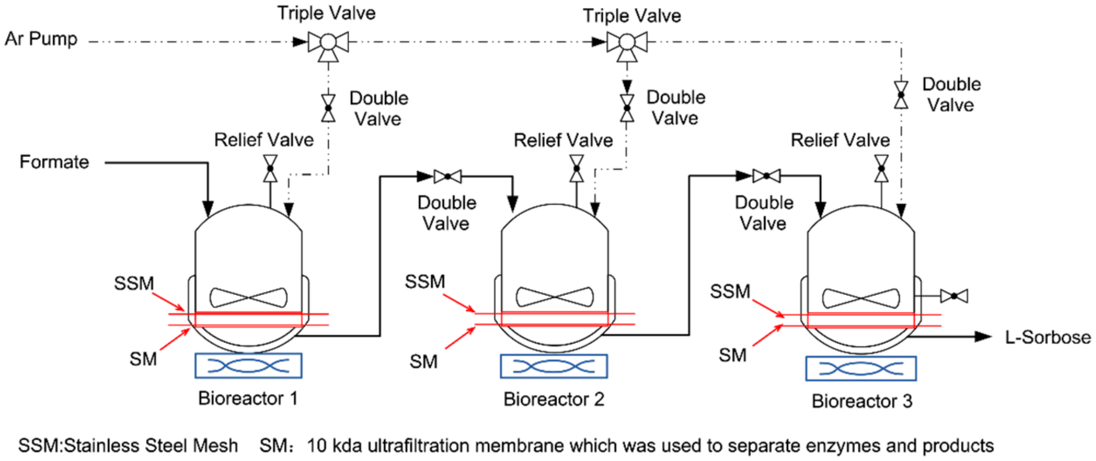

Supplementary Fig. 41 | The structure of designed enzyme cascade reactors. SSM: stainless steel mesh. SM: 10 KDa ultrafiltration membrane.

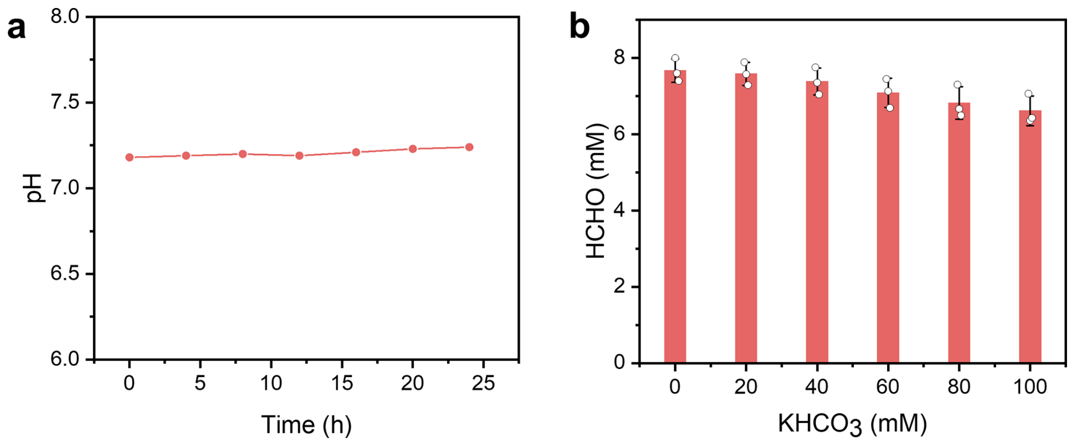

Supplementary Fig. 42 | a, The monitored pH of the reaction solution in CSTR-1 during 24 h of reaction. The data was measured every 4 hours. **b**, The concentration of produced formaldehyde under different concentrations of $KHCO_3$ in CSTR-1. Data points are reported as mean ± standard deviation derived from 3 independent experiments (n = 3).

The effects of the concentrated electrolyte on the pH and enzyme catalysis in CSTR-1 have been investigated. We first monitored the pH in CSTR-1 during the reaction for 24 h using a high-precision pH meter (Mettler Toledo, Zurich, Switzerland). The results showed that the pH was maintained in the range of 7.2 ± 0.1 (Supplementary Fig. 42a), indicating that the continuous input of concentrated electrolyte during the reaction did not significantly affect the pH in CSTR-1. This feature is attributed to the effect of TEA buffer solution, and the concentrated electrolyte will also be diluted in CSTR-1. However, the high concentration of $KHCO_3$ led to a decrease in the yield of HCHO (Supplementary Fig. 42b), most likely because the high concentrations of $KHCO_3$ can reduce the enzyme activity. To avoid the effect of highly concentrated bicarbonate electrolyte on enzyme activity, a porous solid-electrolyte reactor has been employed to directly produce pure HCOOH solution without containing bicarbonate, followed by adding a neutralization module to adjust the pH with cost-effective NaOH.

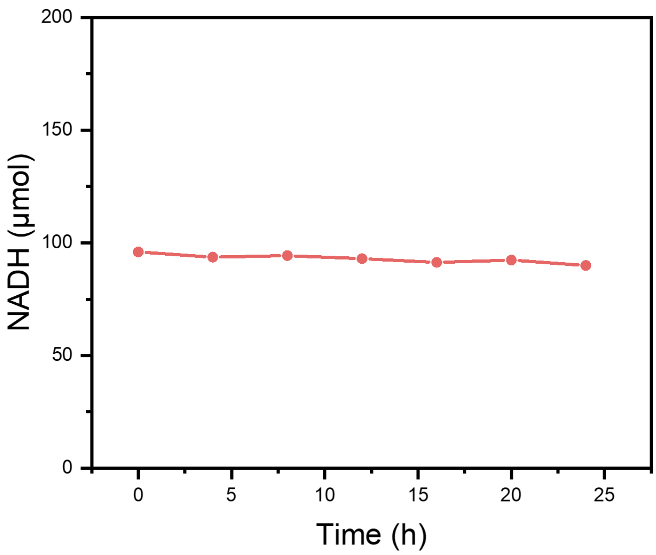

Supplementary Fig. 43 | The changes of NADH concentration in CSTR-1 during the reaction.

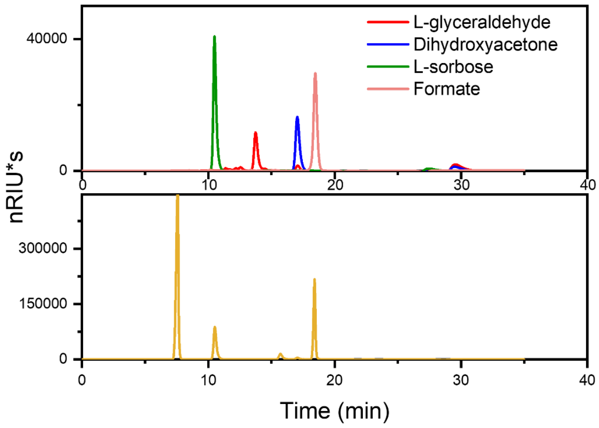

Supplementary Fig. 44 | HPLC analysis of final reaction solution in the solar-driven electrocatalytic–biocatalytic flow system. The top panel is the HPLC chromatogram of the standards for comparing retention time. The bottom panel is the experimental HPLC chromatogram of final reaction solution in CSTR-3. The peak located near 7.6 min is assigned to the TEA buffer.

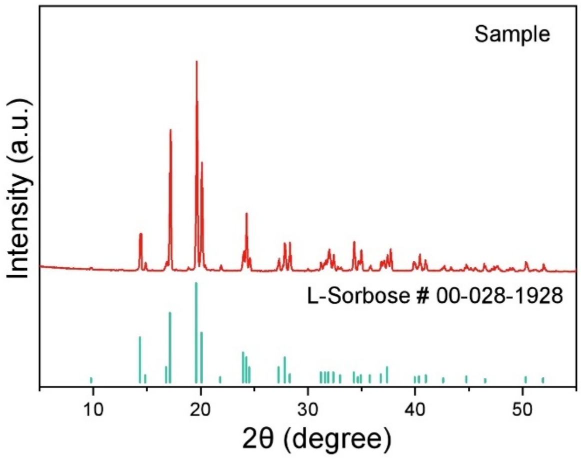

Supplementary Fig. 45 | XRD pattern of produced L-sorbose crystals. The standard diffraction patterns for L-sorbose (JCPDS No. 00-028-1928) is provided as a reference.

XRD pattern for the obtained L-sorbose crystals can be well indexed to the standard diffraction pattern for L-sorbose (JCPDS No. 00-028-1928), indicating the high purity of our L-sorbose crystals directly produced from CO_2.

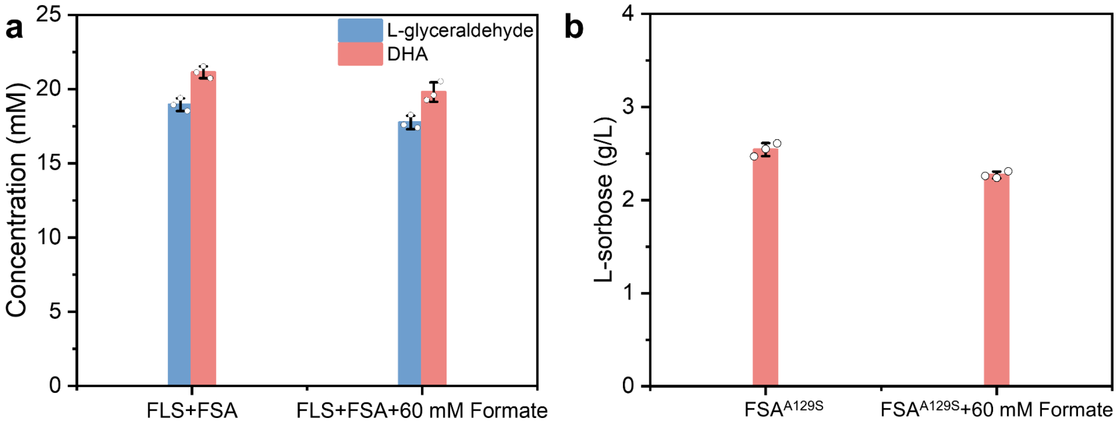

Supplementary Fig. 46 | Effect of residual formate on the product yields after 24 h of enzyme catalytic reaction in each CSTR. **a**, The yields of DHA and L-glyceraldehyde by FLS and FSA catalytic reaction in CSTR-2 in the absence or presence of formate (60 mM). Data points are reported as mean ± standard deviation derived from 3 independent experiments (n = 3). **b**, The yield of L-sorbose yield by FSA^{A129S} catalytic reaction in CSTR-3 in the absence or presence of formate (60 mM). Data points are reported as mean ± standard deviation derived from 3 independent experiments (n = 3).

We have also conducted experiments to explore the effect of the residual formate on enzymatic reaction in CSTR-2 and CSTR-3 (Supplementary Fig. 46). The results demonstrated that the presence of 60 mM (3 mmol) of formate has no significant influence on the downstream enzymatic reactions in CSTR-2 and CSTR-3.

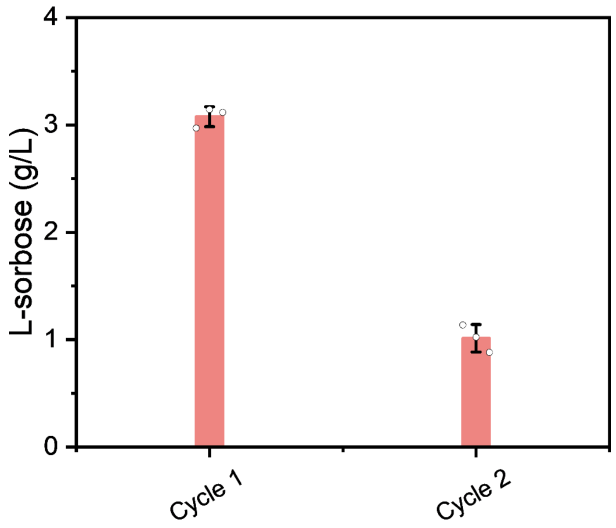

Supplementary Fig. 47 | The yield of L-sorbose by recycling the residual formate solution from CSTR-3 to feed into CSTR-1. The yield of L-sorbose in cycle 2 was calculated by deducting the L-sorbose yield in cycle 1. Data points are reported as mean ± standard deviation derived from 3 independent experiments (n = 3).

We further collected the residual formate solution from CSTR-3 and fed it into CSTR-1 again to continue the enzymatic reaction. The result shows that the residual formate can be converted to L-sorbose with a similar efficiency, indicating that our flow system can fully convert formate to L-sorbose and thus avoid the carbon loss to a great extent.

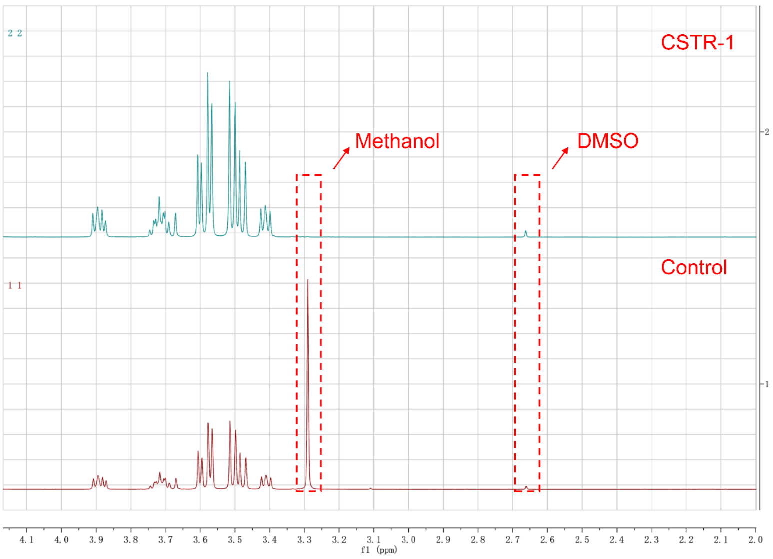

Supplementary Fig. 48 | ^{1}H-NMR spectra of the reaction solution in CSTR-1. The content of methanol was quantitatively analyzed with DMSO as an internal standard. The concentration of methanol in control sample is 50 mM.

The formaldehyde Cannizzaro disproportionation reaction ($2HCHO + OH^- \rightarrow HCOO^- + CH_3OH$) requires the participation of OH^-. We have identified the content of methanol in our system by ^{1}H-NMR spectra (Supplementary Fig. 48). No obvious signal for CH_3OH could be observed in the ^{1}H-NMR spectra. The results indicate that the concentration of methanol was no more than 25 μM (according to the detection limit of CH_3OH by ^{1}H-NMR) in CSTR-1 after 24 h of reaction, most likely because the necessary strong OH^- environment required for the Cannizzaro reaction was not provided. We further tested the effect of methanol on the activity of various enzymes (Supplementary Fig. 49). The results showed that the product yield after 24 h of reaction in each CSTR is not significantly influenced when the concentration of methanol is 25 μM, and is only reduced by 9% when the concentration of methanol increases to 10 mM compared to that without adding methanol. Therefore, the trace amounts of methanol (less than 25 μM) can hardly have a significant negative impact on the overall efficiency of enzyme catalysis in our system. Additionally, considering commercial

formaldehyde often contains methanol as a stabilizer, we have used a methanol-free formaldehyde standard solution (analytical standard, 10 mg/ml in water, Shanghai Aladdin Biochemical Technology Co., Ltd.) as the substrate to avoid the influence of methanol when optimizing and verifying the performance of individual formate-to-formaldehyde conversion module.

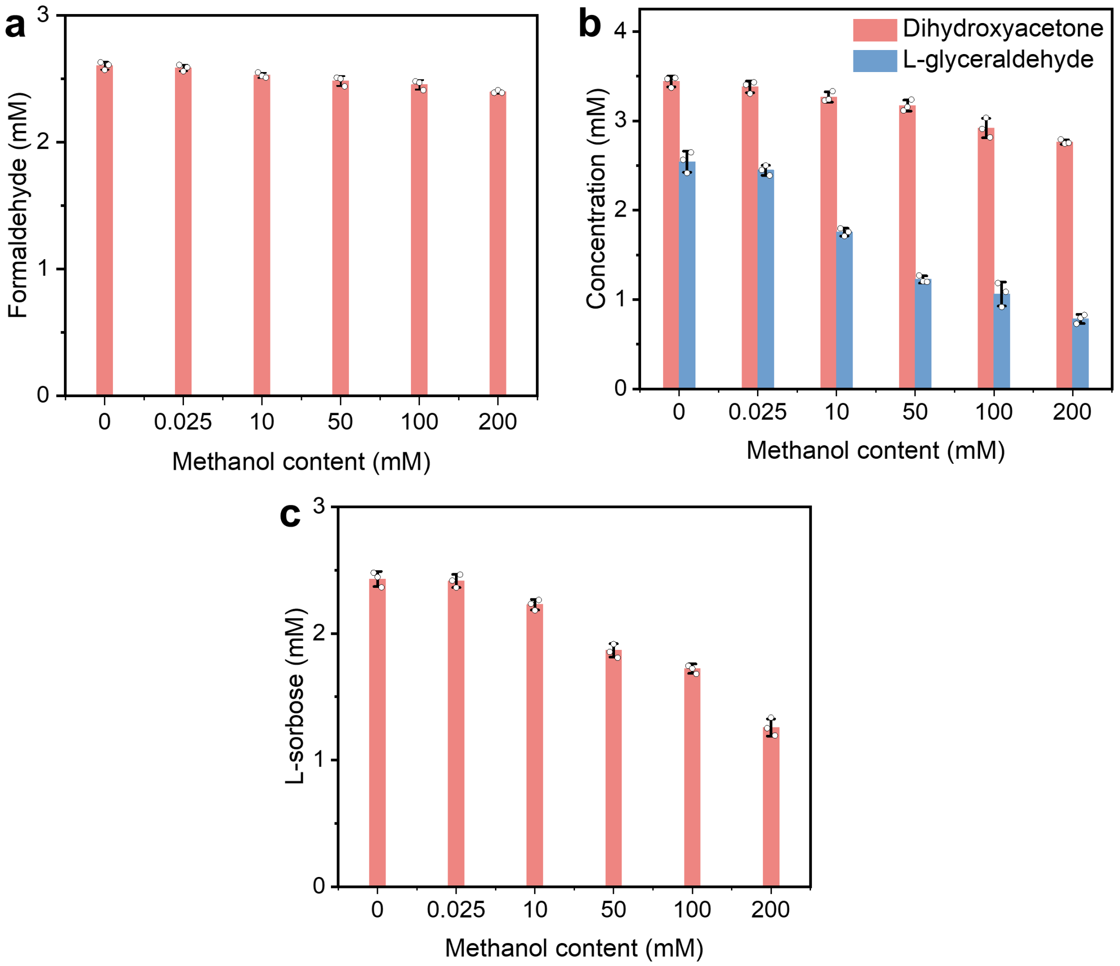

Supplementary Fig. 49 | Effect of different concentrations of methanol on the product yields after 24 h of enzyme catalytic reaction in each CSTR. **a**, The effect of different content of methanol on formaldehyde yield by BmfaldDH and PTDH catalytic reaction in CSTR-1. Data points are reported as mean ± standard deviation derived from 3 independent experiments (n = 3). **b**, The effect of different content of methanol on DHA and L-glyceraldehyde yields by FLS and FSA catalytic reaction in CSTR-2. Data points are reported as mean ± standard deviation derived from 3 independent experiments (n = 3). **c**, The effect of different content of methanol on L-sorbose yield by FSA^{A129S} catalytic reaction in CSTR-3. Data points are reported as mean ± standard deviation derived from 3 independent experiments (n = 3).

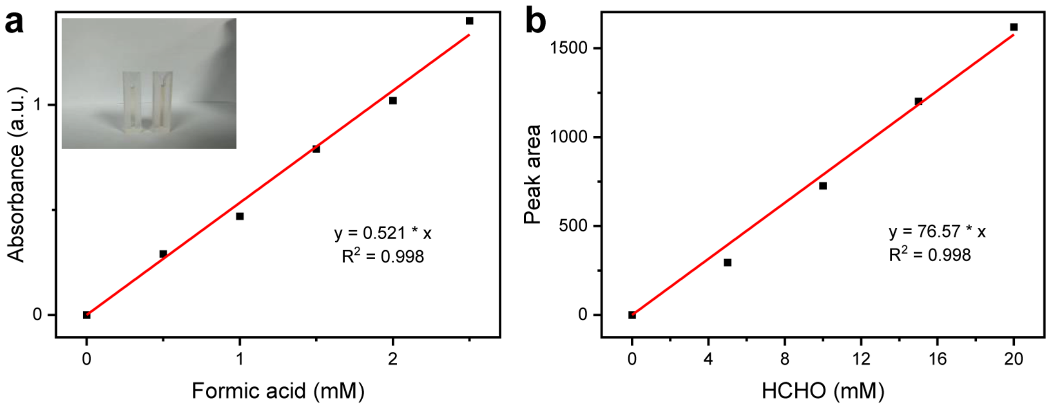

Supplementary Fig. 50 | (a) Calibration curve for quantification of formic acid by Lang & Lang method. (**b**) Calibration curve for quantification of formaldehyde by gas chromatography.

Supplementary Table 1 | Photovoltaic parameters of solar cell measured under one-sun illumination (AM 1.5G, 100 mA cm^{-2}).

Parameter	Value
Open circuit potential (E_{oc})	5.85 V
Short circuit current (I_{sc})	0.15 A
Maximum power (P_{max})	0.60 W
Maximum potential (E_{max})	4.53 V
Maximum current (I_{max})	0.13 A
Fill Factor (FF)	0.69
Efficiency (η)	0.155

Supplementary Table 2 | List of 29 faldDH genes for biochemical screening.

Organism	Size (kb)	Activity (U/mL)	Protein (mg/mL)
Arthrobacter phenanthrenivorans	1.215	≤0.001	4.4
Agrobacterium tumefaciens str. C58	1.197	0.002	4.2
Azoarcus sp. KH32C	1.197	0.006	2.8
Alcanivorax dieselolei B5	1.197	0.005	3.6
Arthrobacter chlorophenolicus	1.212	0.003	3.1
Amycolatopsis mediterranei	1.137	0.002	2.7
Burkholderia mallei	1.200	0.003	2.4
Burkholderia multivorans CF2	1.197	1.02	2.9
Chondromyces crocatus	1.194	ND	3.2
Chromohalobacter salexigens	1.188	ND	3.1
Collimonas fungivorans Ter331	1.203	0.001	3.7
Dyadobacter fermentans	1.188	ND	4.1
Geodermatophilus obscurus	1.254	ND	4.2
Hyphomicrobium zavarzinii	1.403	0.003	4.0
Halopiger xanaduensis	1.167	ND	2.9
Komagataella pastoris	1.098	≤0.001	3.6
Mycobacterium neoaurum	1.215	0.002	4.1
Nakamurella multipartite	1.230	≤0.001	2.5
Pseudomonas fluorescens	1.200	ND	2.7
Ralstonia solanacearum	1.197	0.006	2.6
Rhizobium leguminosarum bv. Viciae	1.191	0.002	2.7
Rhodopirellula baltica	1.209	0.004	3.1
Sulfobacillus acidophilus	1.134	ND	3.8
Salinicoccus halodurans	1.215	ND	3.6
Terriglobus saanensis	1.191	0.002	2.7
Thalassospira xiamenensis	1.191	≤0.001	2.1
Virgibacillus sp.	1.218	ND	1.9
Verminephrobacter eiseniae	1.200	ND	2.8
Verrucosispora maris	1.149	0.002	3.1

Supplementary Table 3 | Comparison of kinetic parameters of various enzymes.

Enzyme	Substrate	K_m (mM)[a]	k_{cat} [s^{-1}][b]	k_{cat}/K_m [mM^{-1} s^{-1}]	Ref.
CcFDH	Sodium bicarbonate	0.370 ± 0.02	2.760 ± 0.21	7.46 ± 0.15	16
PpFaldDH	Formate	ND[c]	ND	ND	17
BmfaldDH	Formate	1.70 ± 0.20	0.653 ± 0.032	0.41 ± 0.05	[d]
PTDH-WT	NAD^+	0.06 ± 0.01	1.03 ± 0.01	18 ± 1	18
PTDH-A155N	NAD^+	0.08 ± 0.01	1.48 ± 0.13	18.5 ± 1	[d]
PTDH-E175A	NAD^+	0.04 ± 0.02	2.84 ± 0.01	71.5 ± 1.2	[d]
PTDH-A176R	NAD^+	0.06 ± 0.01	2.74 ± 0.01	45.7 ± 1.3	[d]
PTDH-Mutant	NAD^+	0.02 ± 0.01	1.85 ± 0.131	92.5 ± 1	[d]
FLS	Formaldehyde	3.55 ± 0.1	17.2 ± 0.41	4.7 ± 0.1	19
FSA	Glycolaldehyde	0.2 ± 0.05	16.5	83	20
FSA	Dihydroxyacetone	32 ± 1	116	4	21
FSA^{A129S}	Dihydroxyacetone	11 ± 1	760	69	21

[a]: K_m (Michaelis constant, $[S]/V=1/V_{max}[S]+K_m/V_{max}$). Plotting [S]/V against [S] yields a line with slope $1/V_{max}$ and an intercept of K_m/V_{max} on the [S]/V axis and $-K_m$ on the [S] axis. [b]: $k_{cat}=(V_{max}/V_{max\ Enzyme}) \times k_{cat\ Enzyme}$. [c]: not detected, [d]: this study.

Supplementary Table 4 | The final contents of various products in each bioreactor at the end of the whole CO_2-to-L-sorbose process by 72 h reaction.

Module	Formate (mmol)	HCHO (mmol)	DHA (mmol)	L-glyceraldehyde (mmol)	L-sorbose (mmol)	By-product[a] (mmol)
PV-EC	10.00	n.d.[b]	n.d.	n.d.	n.d.	n.d.
CSTR-1	3.17	6.55	n.d.	n.d.	n.d.	n.d.
CSTR-2	3.16	0.30	1.13	0.94	n.d.	5×10^{-4}
CSTR-3	3.16	0.07	0.002	0.059	0.86	0.21

[a]: By-product is L-erythrulose ($C_4H_8O_4$). [b]: "n.d." refers to not detected.

When the upstream 10.00 mmol (200 mM) of formate was introduced into CSTR-1, about 65.5% of the formate was converted to produce 6.55 mmol formaldehyde after 24 h reaction, while the content of residual formate in CSTR-1, CSTR-2 and CSTR-3 is around 3.17 mmol. In CSTR-2, about 94.8% of formaldehyde was converted to obtain 1.13 mmol DHA and 0.94 mmol of L-glyceraldehyde, and the content of residual formaldehyde in CSTR-2 is 0.30 mmol. A tiny amount (5×10^{-4} mmol) of the by-product L-erythrulose ($C_4H_8O_4$) was produced due to the addition reaction of DHA and HCHO under the catalysis of FSA. In CSTR-3, 76.1% of DHA and 91.5% of L-glyceraldehyde were converted to produce 0.86 mmol (154.9 mg) of L-sorbose. The reduced content of residual formaldehyde in CSTR-3 was caused by the addition reaction of DHA and HCHO under the catalysis of FSA^{A129S}, producing 0.21 mmol by-product L-erythrulose ($C_4H_8O_4$). As the by-product L-erythrulose is a rare and expensive C_4 sugar, the carbon loss in by-product could be greatly prevented.

Supplementary Table 5 | Primers used in this study.

Primer	Sequence (5'-3')
FSA-F	GGAATTCCATATGCATCATCACCATCACCATGAACTGTATCTGGATACTTCAGAC
FSA-R	ACTCAAGCTTCTATTAAATCGACGTTCTGCCAAA
FSAA129S-F	TGCGGAATATGTTTCGCCTTACGTTAATCGT
FSAA129S-R	AAACATATTCCGCACCTGCCAGCGC
PTDH-W-F	ATGCTGCCTAAGCTTGTCATCACCCACCGA
PTDH-W-R	TCAGCAGGCGGCAGGCTCGGCCTTAGG
PTDH-M-1-F	TGGCATCCTGGGCATGGGTAACATTGGTCTGGCCATGGCTG
PTDH-M-1-R	CCTGAAGTCGATCAGCCATGGCCAGACCAATGTT
PTDH-M-2-F	CCTTCAATACCACGCCTTCAAGGCTCTGGACA
PTDH-M-2-R	GGAAGTTATGGTGCGGAAGTTCCGAGACCTGT
PTDH-M-3-F	CACTGTTGGCATCCTGGGCATGGGTAACTATTGG
PTDH-M-3-R	GTTACCCATGCCCAGGATGCCAACAGTGGCG
PTDH-M-4-F	CTGGGGTGCCACCCTTCAATACCACGCCGCCAA
PTDH-M-4-R	CGGGTGGTATTGAAGGGTGGCACCCCAG
PTDH-M-5-F	CCTTCAATACCACGAGCGAAAGGCTCTG
PTDH-M-5-R	GCTCTCGTGGTATTGAAGGGTGGCACCC
FLS-F	CCATATGATGGCTATGATTACTGGTGGTGAAC
FLS-R	CAAGCTTTCACGCGCCGGATTGGAAATACAGA
Bm-F	CGGGATCCATGAGCAGCAATCGAGGTGTCGTCT
Bm-R	CCTCGAGTCAGGCTGCCAGCAAACCGTGCGGA
Cc-F	CCATATGATGAGCAATGGCATTCATGAAATTGATGA
Cc-R	CCTCGAGTCAGGCTGCCAGCAAACCGTGCGGA
T7	TAATACGACTCACTATAGGG
T7-ter	TGCTAGTTATTGCTCAGCGG

Supplementary Table 6 | Strains and plasmids in this study.

Strains or plasmids	Relevant characteristics, Genotype or Phenotype	Source
E.Coli Strains		
DH5α	*supE44 ΔlacU169 (φ80 lacZΔM15) hsdR17 recA1 endA1 gyrA96 thi-1 relA1*	Novagen
BL21(DE)3	*F-ompT hsdSB (Rb - mB -) gal (λ c I 857 ind1 Sam7 nin5 lacUV5 T7gene1) dcm (DE3)*	Novagen
MG 1655	*F- λ- ilvG- rfb-50 rph-1*	Novagen
Plasmids		
pET 21a (+)	The pET-21a (+) vectors carry an N-terminal T7-Tag sequence plus an optional C-terminal His-Tag sequence	Novagen
pET 28a (+)	The pET-28a (+) vectors carry an N-terminal T7-Tag and His-tag sequence plus an optional C-terminal His-Tag sequence	Novagen
pET 21a-*FLS*	FLS coding sequence cloned into the MCS site of pET 21a	This Study
pET 21a-*FSA*	FSA coding sequence cloned into the MCS site of pET 21a	This Study
pET 21a-FSA^{A129S}	Mutant FSA^{A129S} coding sequence cloned into the MCS site of pET 21a	This Study
pET 28a-*CcFDH*	CcFDH coding sequence cloned into the MCS site of pET 28a	This Study
pET 28a-*BmfaldDH*	BmfaldDH coding sequence cloned into the MCS site of pET 28a	This Study
pET 28a-*PTDH-WT*	PTDH coding sequence cloned into the MCS site of pET 28a	This Study
pET 28a-*PTDH-A155N*	Mutant $PTDH^{A155N}$ coding sequence cloned into the MCS site of pET 28a	This Study
pET 28a-*PTDH-E175A*	Mutant $PTDH^{E175A}$ coding sequence cloned into the MCS site of pET 28a	This Study
pET 28a-*PTDH-A176R*	Mutant $PTDH^{A176R}$ coding sequence cloned into the MCS site of pET 28a	This Study
pET 28a-*PTDH-Mutant*	Mutant $PTDH^{A155N\ E175A\ A176R}$ coding sequence cloned into the MCS site of pET 28a	This Study

Supplementary Table 7 | Key resources used in this study.

Reagent or Resource	Source
PrimeSTAR® HS DNA Polymerase	Takara Bio
PrimeSTAR® Max DNA Polymerase	Takara Bio
Phusion™ High-Fidelity DNA Polymerase	Thermoscientific
Premix Taq™ (Ex Taq™Version 2.0 plus dye)	Takara Bio
SanPrep Column Plasmid Mini-Preps Kit	Sangon Bio
EZ-10 Spin Column Bacterial Genomic DNA Isolation Kit	Sangon Bio
In-Fusion® Snap Assembly EcoDry™ Master Mix Kit	Takara Bio
SanPrep Column DNA Gel Extraction Kit	Sangon Bio
BeyoGold™ His-tag Purification Resin	Beyotime Bio
BeyoGold™ Protease inhibitor mixture	Beyotime Bio
LB Broth Powder	Sangon Bio
Glycerol	Sangon Bio

Supplementary Table 8 | List of amino acid sequences in this study.

Gene	Sequence
CcFDH	MSNGIHEIDDCDLVFIFGYNGADSHPIVARRIVNAKRKGAKIVVTDPRITESARIADLW<br>LPIKNGTNMILVNAFANVLINEGLYNKQYVEEHTVGFEEYKALVEKYTPEYAEKMTG<br>VPAEDIRKSMRMYSKAKNAMILYGMGVCQFGQAVDVVKGLASLALLTGNFGRPNVG<br>IGPVRGQNNVQGACDMGALPNVYPGYQSVTNDAIREKFENAWGVKLPNKVGYHLTE<br>VPHLVLKEDKIKAYYIMGEDPVQSDPDAAEVREALDKLELVIVQDIFMNKTALHADVI<br>LPATSWGEHEGVYSSADRGFQRFRKAIEPTGDVKPDWQIISEIAKAMGYDMNYKNTK<br>EIWDELRNLCPNFKGASYERLEELGGIQWPCPSEDHPGTSYLYKGNKFNTPSGKANLF<br>AAEWRAPMESTDKEYPLVLSTVREVGHYSVRTMTGNCRALQQLADEPGYVQINPED<br>AKNLNILDQEFVRISSRRGSVVAKALVTDRVNKGAVYMTYQWWVGACNELTLNNLD<br>PISKTPEYKYCAVKVENIKDQKAAEQYVQDEYTKIRKKMNINLECCK*
PTDH-WT	MLPKLVITHRVHDEILQLLAPHCELMTNQTDSTLTREEILRRCRDAQAMMAFMPDRV<br>DADFLQACPELRVVGCALKGFDNFDVDACTARGVWLTFVPDLLTVPTAELAIGLAVG<br>LGRHLRAADAFVRSGEFQGWQPQFYGTGLDNATVGILGMGAIGLAMADRLQGWGA<br>TLQYHEAKALDTQTEQRLGLRQVACSELFASSDFILLALPLNADTQHLVNAELLALVR<br>PGALLVNPCRGSVVDEAAVLAALERGQLGGYAADVFEMEDWARADRPRLIDPALLA<br>HPNTLFTPHIGSAVRAVRLEIERCAAQNIIQVLAGARPINAANRLPKAEPAAC*
PTDH-A155N	MLPKLVITHRVHDEILQLLAPHCELMTNQTDSTLTREEILRRCRDAQAMMAFMPDRV<br>DADFLQACPELRVVGCALKGFDNFDVDACTARGVWLTFVPDLLTVPTAELAIGLAVG<br>LGRHLRAADAFVRSGEFQGWQPQFYGTGLDNATVGILGMGNIGLAMADRLQGWGA<br>TLQYHEAKALDTQTEQRLGLRQVACSELFASSDFILLALPLNADTQHLVNAELLALVR<br>PGALLVNPCRGSVVDEAAVLAALERGQLGGYAADVFEMEDWARADRPRLIDPALLA<br>HPNTLFTPHIGSAVRAVRLEIERCAAQNIIQVLAGARPINAANRLPKAEPAAC*
PTDH-E175A	MLPKLVITHRVHDEILQLLAPHCELMTNQTDSTLTREEILRRCRDAQAMMAFMPDRV<br>DADFLQACPELRVVGCALKGFDNFDVDACTARGVWLTFVPDLLTVPTAELAIGLAVG<br>LGRHLRAADAFVRSGEFQGWQPQFYGTGLDNATVGILGMGAIGLAMADRLQGWGA<br>TLQYHAAKALDTQTEQRLGLRQVACSELFASSDFILLALPLNADTQHLVNAELLALVR<br>PGALLVNPCRGSVVDEAAVLAALERGQLGGYAADVFEMEDWARADRPRLIDPALLA<br>HPNTLFTPHIGSAVRAVRLEIERCAAQNIIQVLAGARPINAANRLPKAEPAAC*
PTDH-A176R	MLPKLVITHRVHDEILQLLAPHCELMTNQTDSTLTREEILRRCRDAQAMMAFMPDRV<br>DADFLQACPELRVVGCALKGFDNFDVDACTARGVWLTFVPDLLTVPTAELAIGLAVG<br>LGRHLRAADAFVRSGEFQGWQPQFYGTGLDNATVGILGMGAIGLAMADRLQGWGA<br>TLQYHERKALDTQTEQRLGLRQVACSELFASSDFILLALPLNADTQHLVNAELLALVR<br>PGALLVNPCRGSVVDEAAVLAALERGQLGGYAADVFEMEDWARADRPRLIDPALLA<br>HPNTLFTPHIGSAVRAVRLEIERCAAQNIIQVLAGARPINAANRLPKAEPAAC*
PTDH-Mutant	MLPKLVITHRVHDEILQLLAPHCELMTNQTDSTLTREEILRRCRDAQAMMAFMPDRV<br>DADFLQACPELRVVGCALKGFDNFDVDACTARGVWLTFVPDLLTVPTAELAIGLAVG<br>LGRHLRAADAFVRSGEFQGWQPQFYGTGLDNATVGILGMGNIGLAMADRLQGWGA<br>TLQYHARKALDTQTEQRLGLRQVACSELFASSDFILLALPLNADTQHLVNAELLALVR<br>PGALLVNPCRGSVVDEAAVLAALERGQLGGYAADVFEMEDWARADRPRLIDPALLA

	HPNTLFTPHIGSAVRAVRLEIERCAAQNIIQVLAGARPINAANRLPKAEPAAC*
BmfaldDH	MSSNRGVVYLGPGKVEVQKIDYPKMVDPSGRAIGHGVILKVVSTNICGSDQHMVRG<br>RTTAPVGLVLGHEITGEVVEVGRDVETLKIGDLVSVPFNVACGRCAMCKETHTGVCL<br>NVNPSRAGGAYGYVDMGGWIGGQAEYVLVPYADFNLLKFPDRDQAMAKIRDLTCLS<br>DILPTGYHGAVSAGVKPGSTVYIAGAGPVGMAAAASARLLGAAVTIVGDMNAERLA<br>HAKAMGFETVDLSKDATLGEQIAQILGKPEIDCAVDCVGFEAHGHGSSGHAEEAPATV<br>LNSLMEITRPAGAIGIPGLYVTDDPGAQDKAAQHGSLSIRFGLGWAKSHSFFTGQTPVL<br>KYNRNLMQAILYDRLPIAKIVNVTVISLDDAPEGYKKFDGGAPRKFVIDPHGLLAA*
FLS	MAMITGGELVVRTLIKAGVEHLFGLHGIHIDTIFQACLDHDVPIIDTRHEAAAGHAAE<br>GYARAGAKLGVALVTAGGGFTNAVTPIANARTDRTPVLFLTGSGALRDDETNTLQAGI<br>DQVAMAAPITKWAHRVMATEHIPRLVMQAIRAALSAPRGPVLLDLPWDILMNQIDED<br>SVIIPDLVLSAHGAHPDPADLDQALALLRKAERPVIVLGSEASRTARKTALSAFVAATG<br>VPVFADYEGLSMLSGLPDAMRGGLVQNLYSFAKADAAPDLVLMLGARFGLNTGHGS<br>GQLIPHSAQVIQVDPDACELGRLQGIALGIVADVGGTIEALAQATAQDAAWPDRGDW<br>CAKVTDLAQERYASIAAKSSSEHALHPFHASQVIAKHVDAGVTVVADGGLTYLWLSE<br>VMSRVKPGGFLCHGYLNSMGVGFGTALGAQVADLEAGRRTILVTGDGSVGYSIGEFD<br>TLVRKQLPLIVIIMNNQSWGWTLHFQQLAVGPNRVTGTRLENGSYHGVAAAFGADGY<br>HVDSVESFSAALAQALAHNRPACINVAVALDPIPPEELILIGMDPFAGSTENLYFQSGA*
FSA	MELYLDTSDVVAVKALSRIFPLAGVTTNPSIIAAGKKPLDVVLPQLHEAMGGQGRLFA<br>QVMATTAEGMVNDALKLRSIIADIVVKVPVTAEGLAAIKMLKAEGIPTLGTAVYGAA<br>QGLLSALAGAEYVAPYVNRIDAQGGSGIQTVTDLHQLLKMHAPQAKVLAASFKTPR<br>QALDCLLAGCESITLPLDVAQQMISYPAVDAAVAKFEQDWQGAFGRTSI*
FSA^{A129S}	MELYLDTSDVVAVKALSRIFPLAGVTTNPSIIAAGKKPLDVVLPQLHEAMGGQGRLFA<br>QVMATTAEGMVNDALKLRSIIADIVVKVPVTAEGLAAIKMLKAEGIPTLGTAVYGAA<br>QGLLSALAGAEYVSPYVNRIDAQGGSGIQTVTDLHQLLKMHAPQAKVLAASFKTPR<br>QALDCLLAGCESITLPLDVAQQMISYPAVDAAVAKFEQDWQGAFGRTSI*

Reference

1. Desmons, S. *et al.* Enantioselective reductive oligomerization of carbon dioxide into L-erythrulose via a chemoenzymatic catalysis. *J. Am. Chem. Soc.* **143**, 16274-16283 (2021).
2. Lang, E. & Lang, H. Spezifische rarbreaktion zum direkten nachweis der ameisensäure. *Fresenius' Zeitschrift für analytische Chemie* **260**, 8-10 (1972).
3. Yang, B. J. *et al.* A room-temperature route to bismuth nanotube arrays. *Eur. J. Inorg. Chem.*, 3699-3702 (2003).
4. Zheng, T. T. *et al.* Upcycling CO_2 into energy-rich long-chain compounds via electrochemical and metabolic engineering. *Nat. Catal.* **5**, 388-396 (2022).
5. Schreier, M. *et al.* Solar conversion of CO_2 to CO using earth-abundant electrocatalysts prepared by atomic layer modification of CuO. *Nat. Energy* **2**, 17087 (2017).
6. Zeng, G. *et al.* Reconstruction of ultrahigh-aspect-ratio crystalline bismuth–organic hybrid nanobelts for selective electrocatalytic CO_2 reduction to formate. *Adv. Funct. Mater.* **32**, 2201125 (2022).
7. Xia, C. *et al.* Continuous production of pure liquid fuel solutions via electrocatalytic CO_2 reduction using solid-electrolyte devices. *Nat. Energy* **4**, 776-785 (2019).
8. Ma, W. *et al.* Electrocatalytic reduction of CO_2 to ethylene and ethanol through hydrogen-assisted C–C coupling over fluorine-modified copper. *Nat. Catal.* **3**, 478-487 (2020).
9. Fan, L. *et al.* Strategies in catalysts and electrolyzer design for electrochemical CO_2 reduction toward C_{2+} products. *Sci. Adv.* **6**, eaay3111 (2020).
10. Lin, L. *et al.* A nanocomposite of bismuth clusters and $Bi_2O_2CO_3$ Sheets for Highly Efficient Electrocatalytic Reduction of CO2 to Formate. *Angew. Chem. Int. Ed.* **62**, e202214959 (2023).
11. Zhang, L. Y. *et al.* Directed evolution of phosphite dehydrogenase to cycle noncanonical redox cofactors via universal growth selection platform. *Nat. Commun.* **13**, 5021 (2022).
12. Woodyer, R., Wheatley, J. L., Relyea, H. A., Rimkus, S. & van der Donk, W. A. Site-directed mutagenesis of active site residues of phosphite dehydrogenase. *Biochemistry* **44**, 4765-4774 (2005).
13. Woodyer, R., van der Donk, W. A. & Zhao, H. M. Relaxing the nicotinamide cofactor specificity of phosphite dehydrogenase by rational design. *Biochemistry.* **42**, 11604-11614 (2003).
14. Zou, Y. Z. *et al.* Crystal structures of phosphite dehydrogenase provide insights into nicotinamide cofactor regeneration. *Biochemistry.* **51**, 4263-4270 (2012).
15. Woodyer, R., Zhao, H. M. & van der Donk, W. A. Mechanistic investigation of a highly active phosphite dehydrogenase mutant and its application for NADPH regeneration. *Febs Journal* **272**, 3816-3827 (2005).
16. Kuk, S. K. *et al.* Photoelectrochemical reduction of carbon dioxide to methanol through a highly efficient enzyme cascade. *Angew. Chem. Int. Ed.* **56**, 3827-3832 (2017).
17. Ando, M. *et al.* Formaldehyde dehydrogenase from pseudomonas-putida-Purification and some properties. *J. Biochem.* **85**, 1165-1172 (1979).
18. Johannes, T. W., Woodyer, R. D. & Zhao, H. M. Directed evolution of a thermostable phosphite dehydrogenase for NAD(P)H regeneration. *Appl. Environ. Microb.* **71**, 5728-5734 (2005).
19. Siegel, J. B. *et al.* Computational protein design enables a novel one-carbon assimilation pathway. *Proc. Natl Acad. Sci. USA* **112**, 3704-3709 (2015).
20. Garrabou, X. *et al.* Asymmetric self- and cross-aldol reactions of glycolaldehyde catalyzed by D-fructose-6-phosphate aldolase. *Angew. Chem. Int. Ed.* **48**, 5521-5525 (2009).

21. Castillo, J. A. *et al.* A mutant D-fructose-6-phosphate aldolase (Ala129Ser) with improved affinity towards dihydroxyacetone for the synthesis of polyhydroxylated compounds. *Adv. Synth. Catal.* **352**, 1039-1046 (2010).
